# Supplementary material for: Temporally-coordinated bivalent histone modifications of BCG1 enable fungal invasion and immune evasion
Source: Nat Commun. 2024 Jan 5;15:231. doi: 10.1038/s41467-023-44491-6 (PMC10770383; doi:10.1038/s41467-023-44491-6)
Supplement: Supplementary file 1 — supplementary information [file 41467_2023_44491_MOESM1_ESM.pdf]

Supplementary Information

Temporally-coordinated bivalent histone modifications of *BCG1* enable fungal invasion and immune evasion

Xiaozhen Zhao[#1], Yiming Wang[#1], Bingqin Yuan[#1], Hanxi Zhao[#1], Yujie Wang[1], Zheng Tan[1], Zhiyuan Wang[1], Huijun Wu[1], Gang Li[1], Wei Song[1], Ravi Gupta[2], Kenichi Tsuda[3], Zhonghua Ma[4], Xuewen Gao[1], and Qin Gu*[1]

[1]Department of Plant Pathology, College of Plant Protection, Nanjing Agricultural University, Key Laboratory of Monitoring and Management of Crop Diseases and Pest Insects, Ministry of Education, Nanjing, China.

[2]College of General Education, Kookmin University, Seoul 02707, South Korea.

[3]State Key Laboratory of Agricultural Microbiology, Hubei Hongshan Laboratory, Hubei Key Lab of Plant Pathology, College of Plant Science and Technology, Huazhong Agricultural University, Wuhan 430070, China.

[4]State Key Laboratory of Rice Biology, the Key Laboratory of Biology of Crop Pathogens and Insects, Institute of Biotechnology, Zhejiang University, Hangzhou, China.

Contents:

I. **Supplementary Figures**

II. **Supplementary Tables**

I. Supplementary Figures

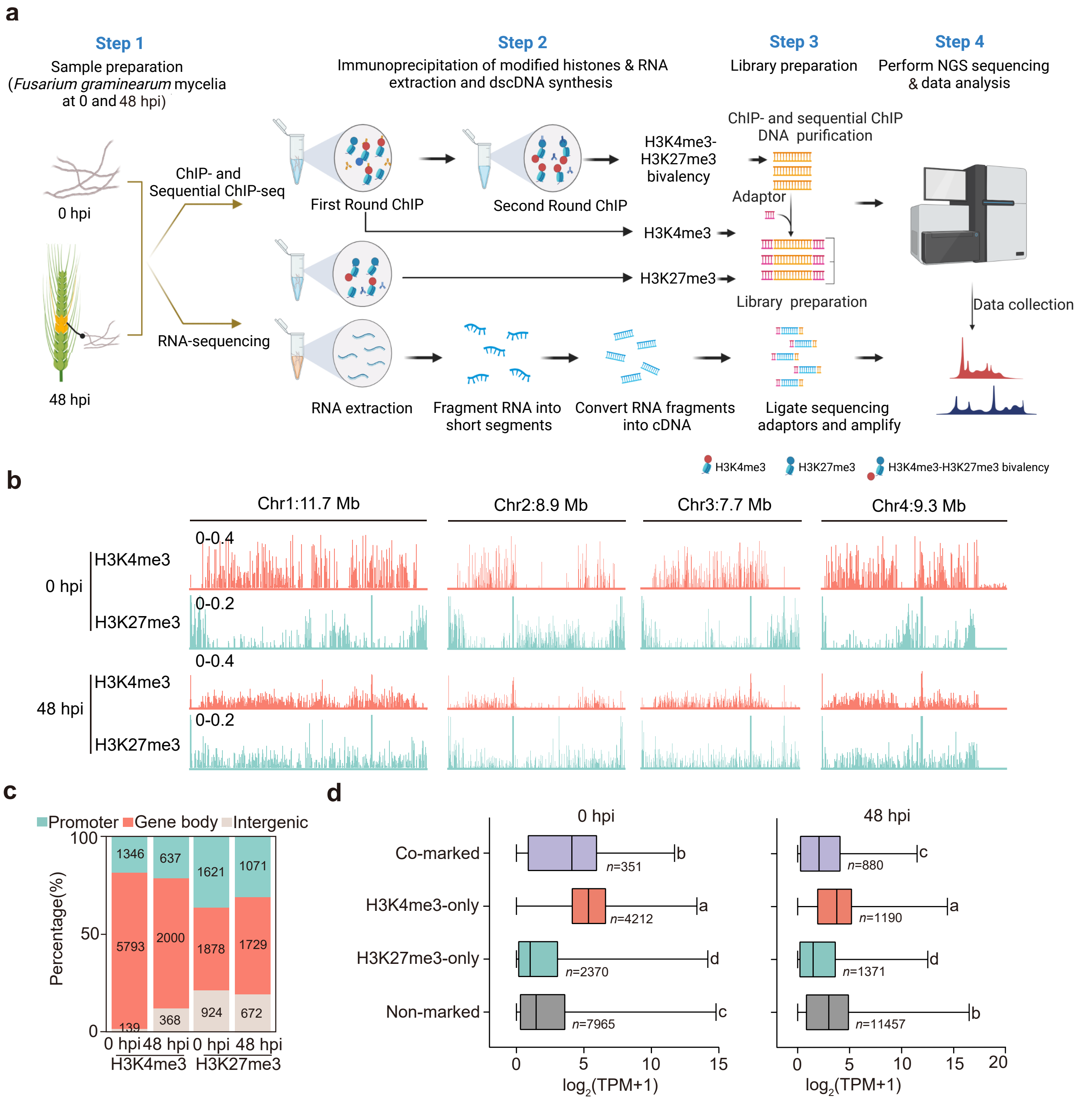

Supplementary Fig. 1 Genome-wide landscapes of H3K4me3 and H3K27me3 modifications in *F. graminearum* during infection. a Schematic diagram of RNA-seq and ChIP-seq analysis of *F. graminearum* samples at 0 and 48 hours post-inoculation (hpi). Figure created using BioRender (http://biorender.com/). **b** ChIP-seq signals of H3K4me3 (red) and H3K27me3 (green) within chromosomes of *F. graminearum* at 0 and 48 hours post-inoculation (hpi) are shown in the IGV browser. The Y-axis represents the ChIP-seq signal density, which was calculated by using BPM (Bins Per Million mapped reads) with 50-bp resolution. **c** Genomic distribution of H3K4me3 and H3K27me3 ChIP-seq peaks for *F. graminearum* at 0 and 48 hpi. **d** Box plots showing the average expression levels of genes marked by different histone modifications. The center line indicates the median; the upper and lower bounds indicate the 75th and 25th percentiles, respectively; and the whiskers indicate the minimum and maximum. The numbers indicate the genes associated with different chromatin states. Different letters denote significant differences (*p*-value < 0.05, one-way ANOVA). Adjusted *p*-values are shown in the Source Data.

a

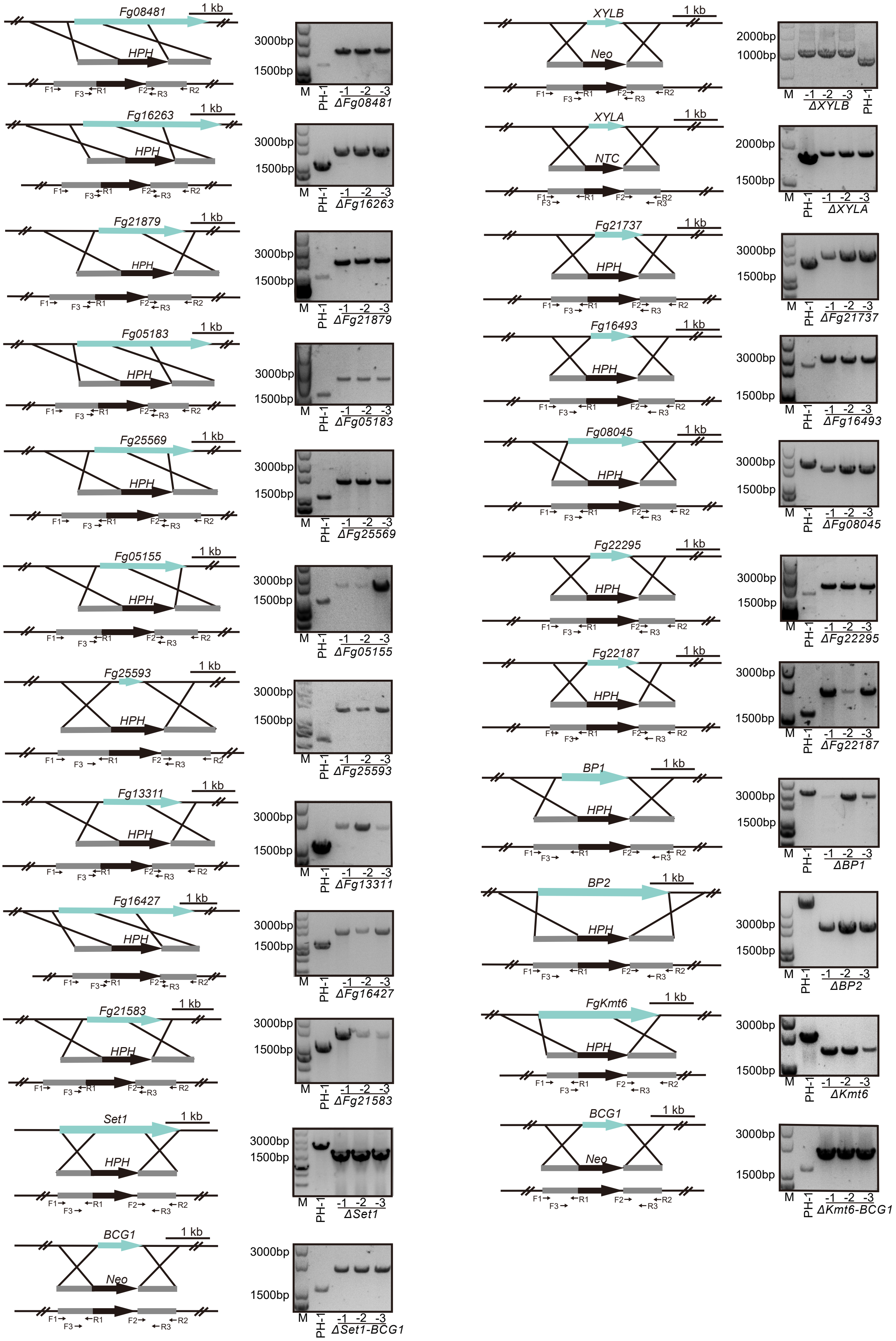
Fg08481
1 kb
HPH
F1 F3 R1 F2 R3 R2
3000bp
1500bp
M PH-1 -1 -2 -3
ΔFg08481
Fg16263
ΔFg16263
Fg21879
ΔFg21879
Fg05183
ΔFg05183
Fg25569
ΔFg25569
Fg05155
ΔFg05155
Fg25593
ΔFg25593
Fg13311
ΔFg13311
Fg16427
ΔFg16427
Fg21583
ΔFg21583
Set1
ΔSet1
BCG1
Neo
ΔSet1-BCG1
XYLB
2000bp
1000bp
ΔXYLB
XYLA
NTC
ΔXYLA
Fg21737
ΔFg21737
Fg16493
ΔFg16493
Fg08045
ΔFg08045
Fg22295
ΔFg22295
Fg22187
ΔFg22187
BP1
ΔBP1
BP2
ΔBP2
FgKmt6
ΔKmt6
ΔKmt6-BCG1

b

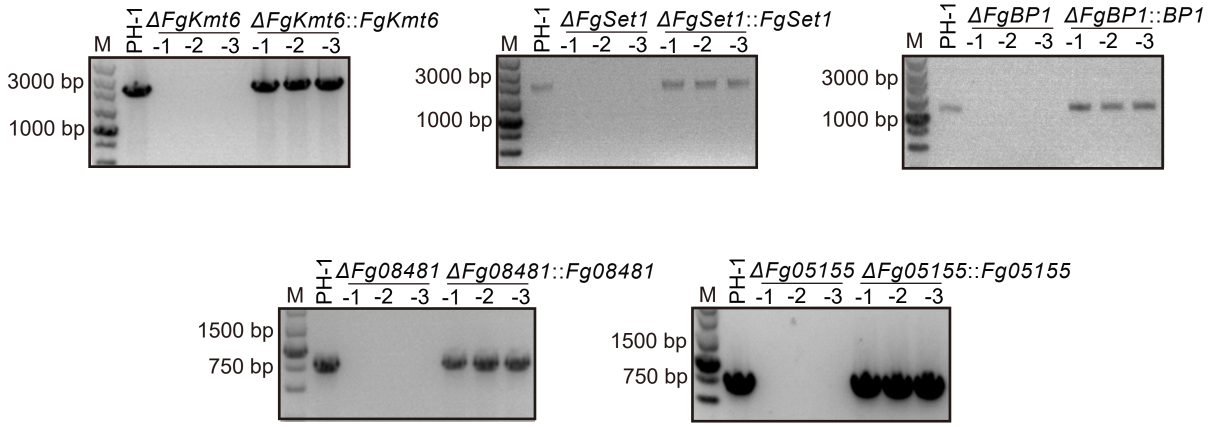

Supplementary Fig. 2 Construction and identification of gene deletion mutants. a Gene replacement strategy for the deletion mutants. The hygromycin resistance cassette (*HPH*) is denoted by the large black arrow (left panel). PCR identification of the wild-type PH-1 and the indicated mutants (right panel). **b** PCR identification of the wild-type PH-1 and complemented strains. M, Marker.

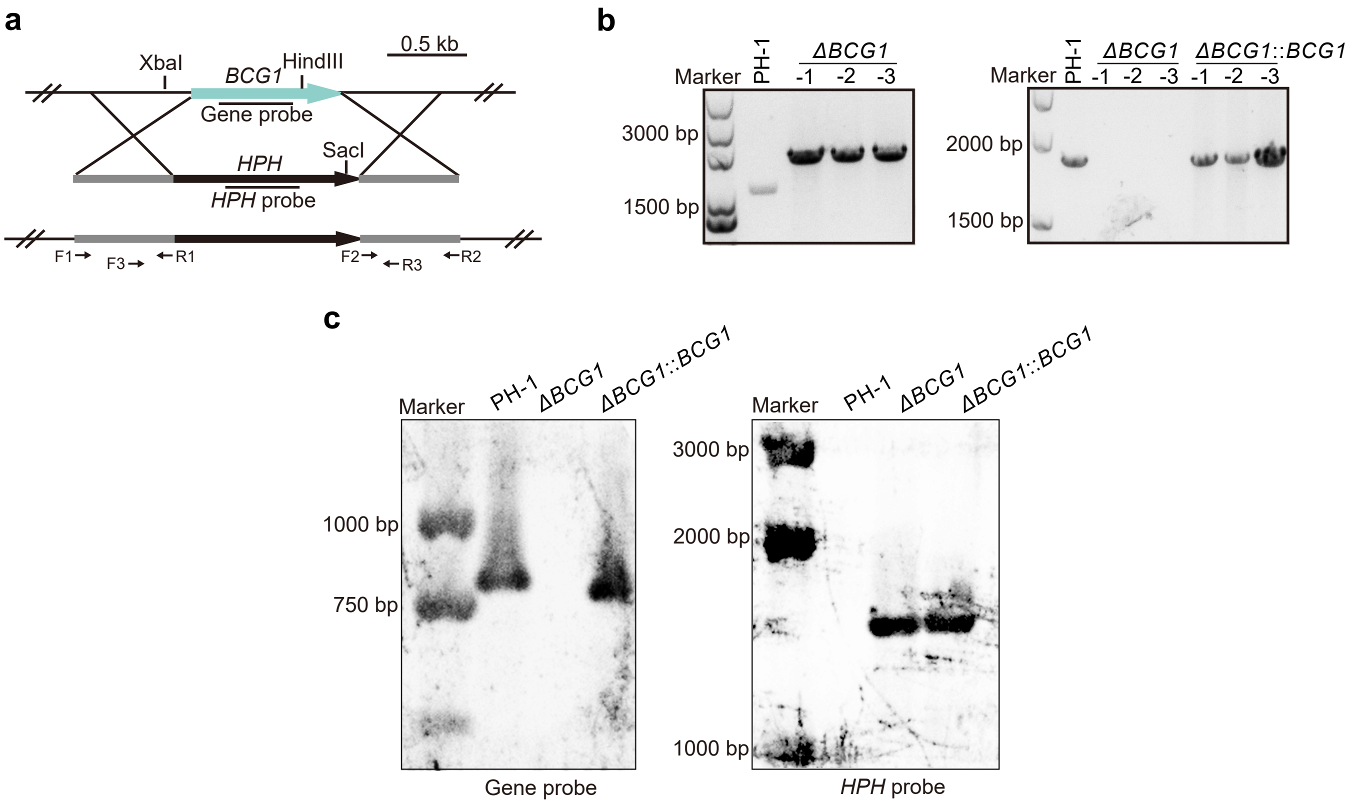

Supplementary Fig. 3 Construction and identification of gene deletion and complemented mutants. a Gene replacement strategy for the deletion mutant *ΔBCG1*. The hygromycin resistance cassette (*HPH*) is denoted by the large black arrow. **b** PCR identification of the wild-type PH-1, the deletion mutants (*ΔBCG1*), and complemented strains (*ΔBCG1*::*BCG1*). **c** Southern blot hybridization analysis of the wild-type PH-1, the deletion mutants (*ΔBCG1*), and complemented strains (*ΔBCG1*::*BCG1*).

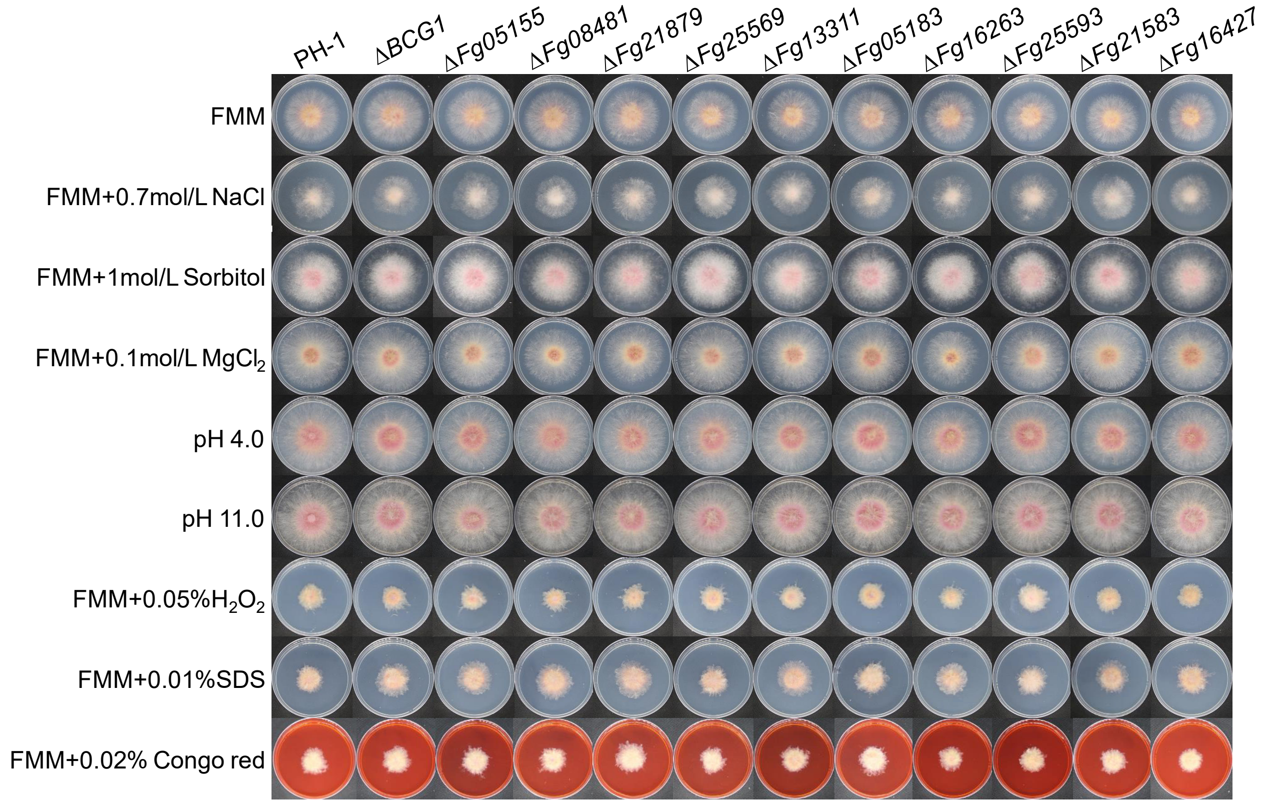

Supplementary Fig. 4 Assays for filamentous growth and stress responses of the 11 *BCGs* mutants. *F. graminearum* wild-type strain PH-1 and the 11 *BCGs* mutants were cultured on FMM supplemented with or without indicated agents at 25°C for 3 days.

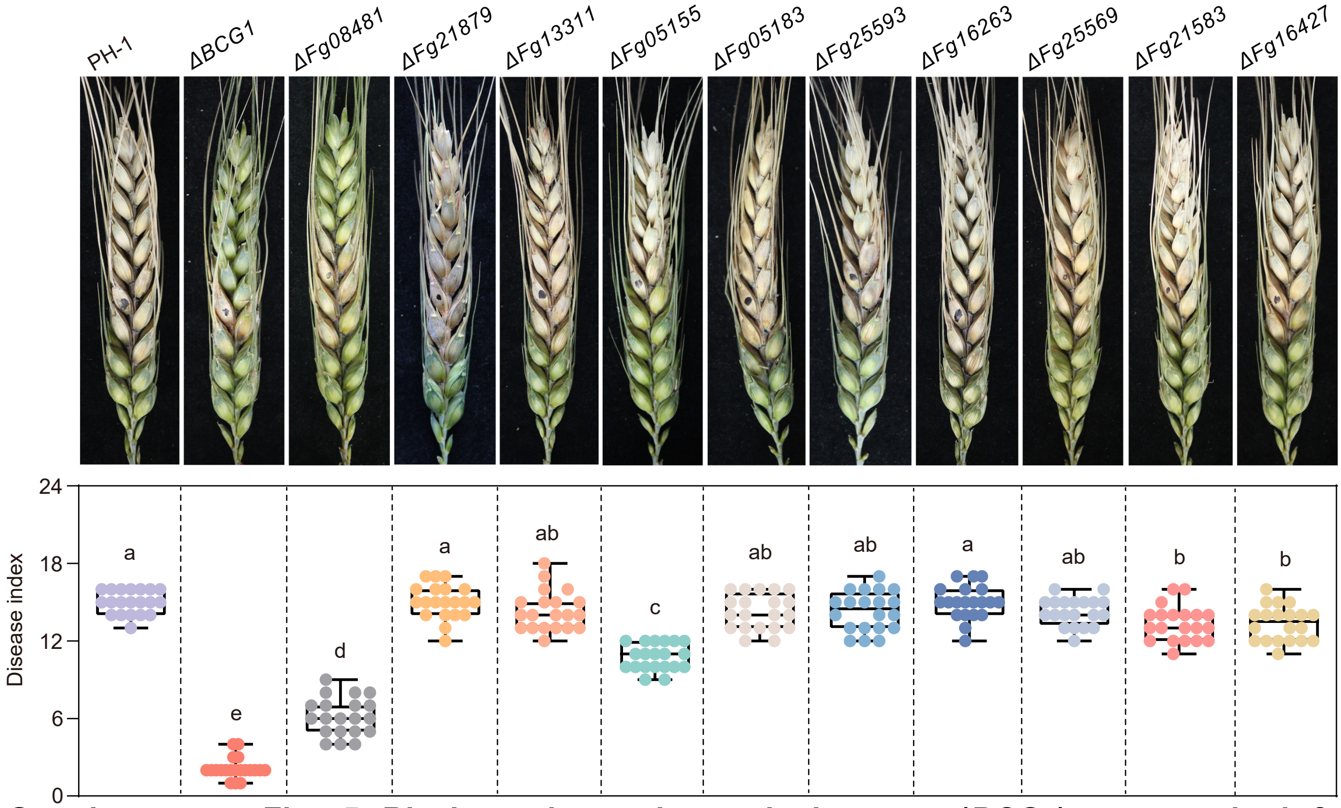

Supplementary Fig. 5 Bivalent chromatin marked genes (*BCGs*) are required for pathogenesis of *F. graminearum*. Virulence of *F. graminearum* wild-type strain PH-1 and the deletion mutants of bivalent chromatin marked genes (*BCGs*) were evaluated on wheat heads (upper panel). Representative images of infected wheat heads were photographed at 14 days post-inoculation (dpi). Quantification was estimated with data obtained from 20 biologically independent samples (lower panel). Different letters denote significant differences (*p*-value < 0.01, one-way ANOVA). *P*-values are shown in the Source Data.

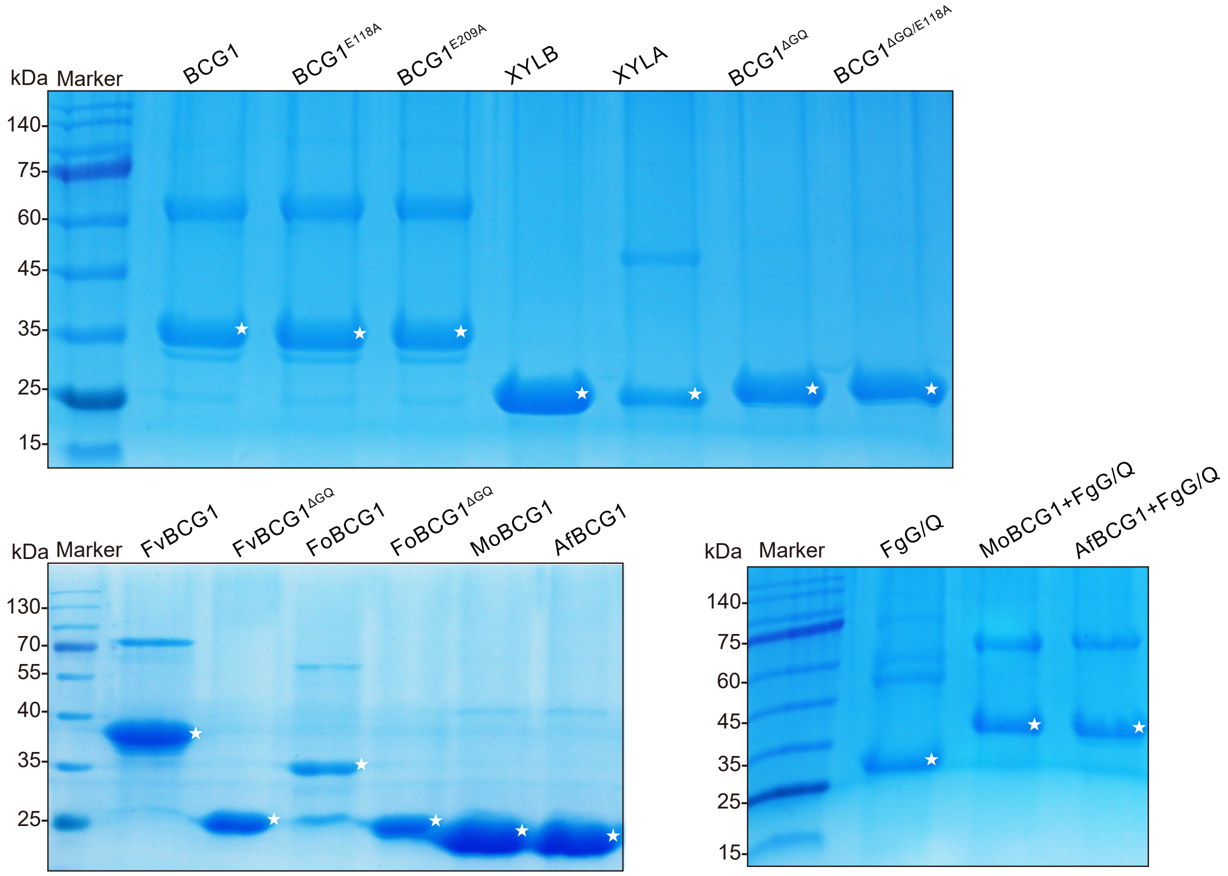

Supplementary Fig. 6 Expression and purification of His-tagged proteins in *P. pastoris*. Fusion proteins containing a 6×His tag at the N terminus were expressed in *p. pastoris* X-33 upon induction for 48 hours at 30°C in the presence of 0.5%, methyl alcohol, and then purified by Ni-NTA affinity chromatography. Coomassie Brilliant Blue G-250 staining showed the protein samples resolved by SDS-PAGE.

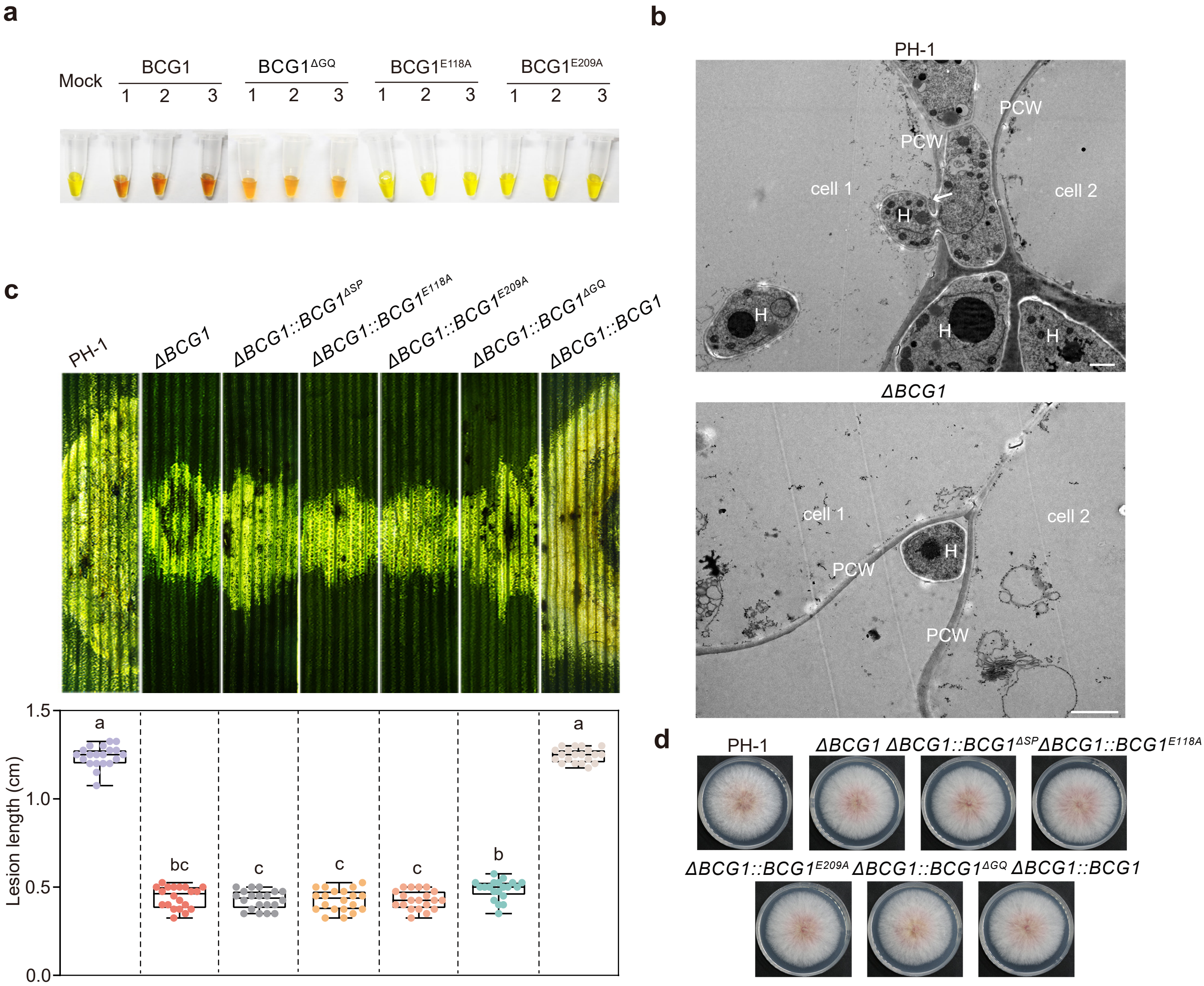

Supplementary Fig. 7 BCG1 is required for the full virulence of *F. graminearum*. a. BCG1 protein is able to degrade the xylan derived from cell wall of wheat palea. Xylan-degrading activity of indicated proteins was determined using the 2,4-dinitrosalicyclic acid (DNS) assay. **b** Transmission electron microscopy (TEM) showed that *ΔBCG1* mutant was unable to breach cell walls of wheat palea at 2 days post-inoculation (dpi). White arrowheads indicate hyphal penetration of the wheat cell wall. PCW: plant cell wall, H: *F. graminearum* hyphae. White arrowheads indicate hyphal penetration of the wheat cell wall. PCW: plant cell wall; H: *F. graminearum* hyphae. Scale bars represent 2 μm. **c** Virulence of PH-1, and its variants on wheat seedling leaves that were inoculated with the fresh mycelial plugs of the indicated *F. graminearum* strains. Representative images of infected wheat seedling leaves were photographed at 3 dpi (upper panel). Lesion lengths of wheat leaves were measured and the data presented are the means ± standard deviation (*n*=20, biologically independent samples). Different letters indicate significant differences (*p*-value <0.01, one-way ANOVA). *P*-values are shown in the Source Data. **d** *F. graminearum* wild-type strain PH-1 and its variants (*ΔBCG1*, *ΔBCG1*::*BCG1*, *ΔBCG1*::*BCG1*^{ΔSP}, *ΔBCG1*::*BCG1*^{ΔGQ}, *ΔBCG1*::*BCG1*^{E118A}, and *ΔBCG1*::*BCG1*^{E209A}) were cultured on PDA at 25°C for 3 days.

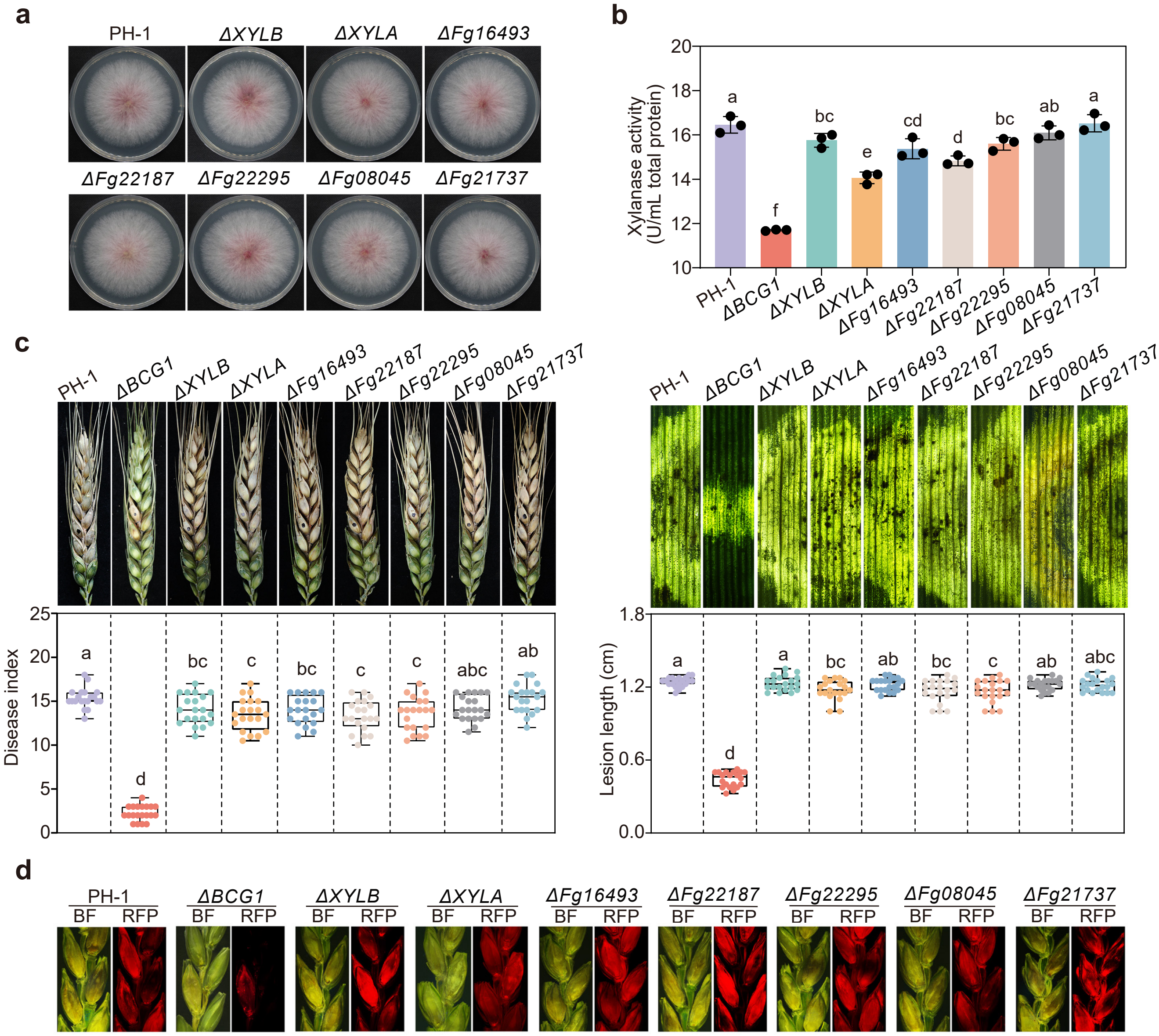

Supplementary Fig. 8 Effects of xylanase genes deletion on filamentous growth, extracellular xylanase activity, and fungal virulence. a PH-1 and the indicated deletion mutants were cultured on PDA at 25°C for 3 days. **b** Each strain was cultured in 10 mL 1.1% CMC medium, and xylanase activity was assayed by a 2,4-dinitrosalicyclic acid (DNS) assay. The values are the means ± standard deviation (*n*=3, biologically independent experiments). Different letters indicate significant differences (*p*-value < 0.01, one-way ANOVA). **c** Virulence of PH-1 and the xylanase mutants were evaluated on wheat heads and seedling leaves. Representative images of infected wheat heads (left panel) and seedling leaves (right panel) were photographed at 14 days post-inoculation (dpi) and 3 dpi, respectively. Quantification was estimated with data obtained from 20 biologically independent samples. Different letters represent significant differences statistically according to the one-way ANOVA followed by Fisher's LSD test (*p*-value < 0.01). *P*-values for **b**, **c** are shown in the Source Data. **d** Cross-sections of inoculated and adjacent wheat spikelets that were inoculated with PH-1 and the mutants bearing FgActin-RFP. The samples were taken at 7 dpi. Abbreviations: BF, bright field; RFP, red fluorescent protein.

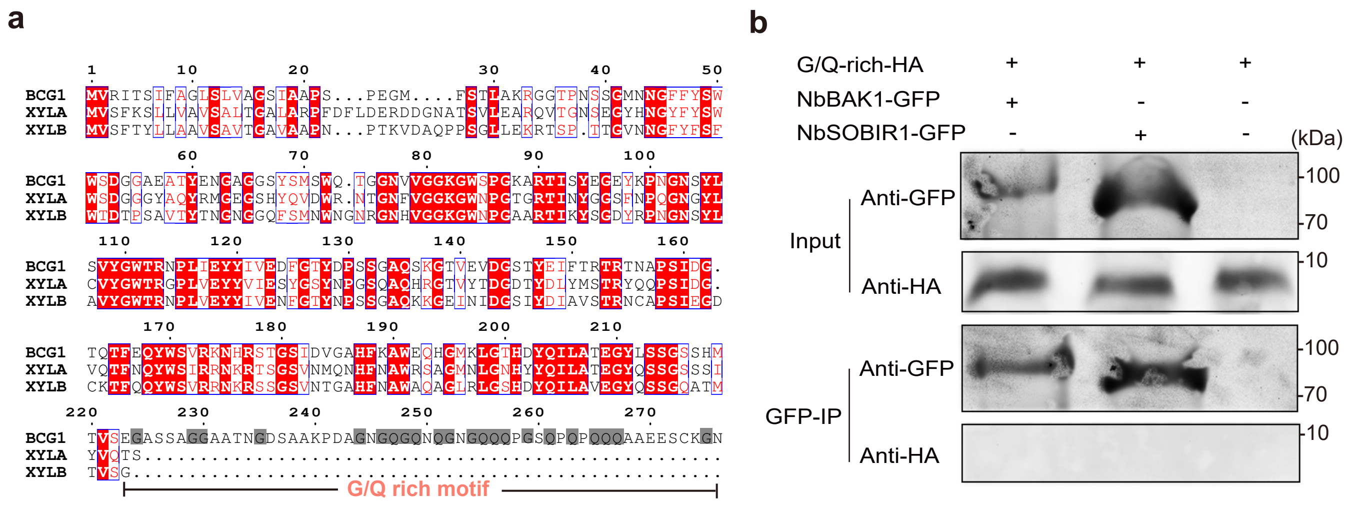

Supplementary Fig. 9 *F. graminearum* G/Q-rich motif fails to associate with NbBAK1 and NbSOBIR1. a Sequence comparison showed that BCG1 contains a previously unrecognized C-terminal G/Q-rich motif. Amino acid alignment of BCG1, XYLA, and XYLB protein sequences using ClustalW, and visualizing with ESPript 3.0. **b** HA-tagged G/Q-rich motif was co-expressed with GFP-tagged NbSORIR1 or NbBAK1 in *N. benthamiana* leaves. Total proteins were extracted at 2 days post-inoculation, and the immune complexes were pulled down using GFP-trap A beads and immunoblotted with anti-HA or anti-GFP antibodies.

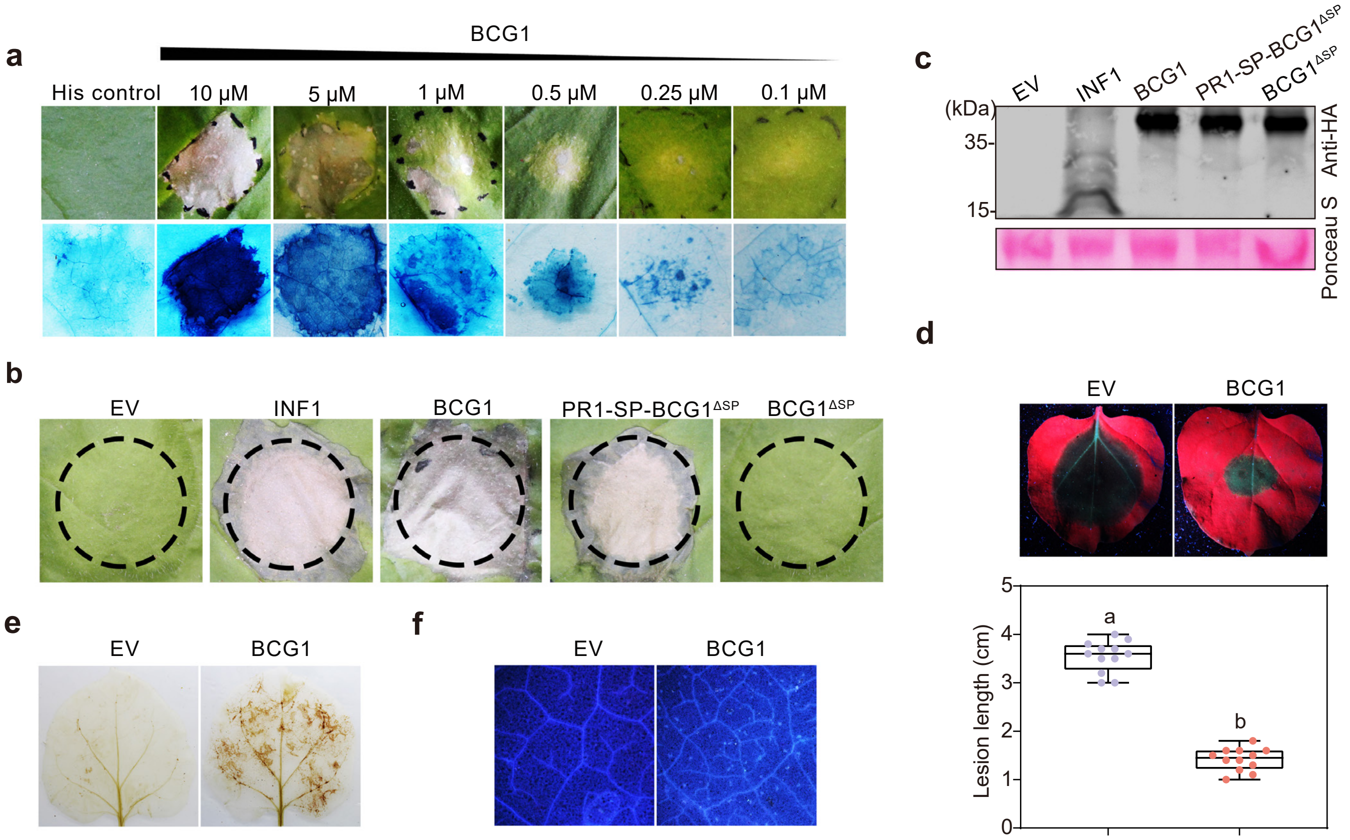

Supplementary Fig. 10 BCG1 triggers immune responses in plants. a *N. benthamiana* leaves were infiltrated with purified BCG1 protein (10 μM to 0.1 μM) or the His control. Representative *N. benthamiana* leaves showing cell death were photographed at 3 dpi (upper panel), and then stained with trypan blue (lower panel). **b** Representative *N. benthamiana* leaves showing cell death at 5 days post-inoculation (dpi) with *Agrobacterium* strains expressing indicated genes in the PVX vector. **c** Western blot analysis of the indicated proteins expressed in *N. benthamiana* at 2 dpi using an anti-HA antibody (upper panel). Protein loading is indicated by Ponceau S staining (lower panel). **d** Disease resistance induced by transient expression of BCG1-HA protein in *N. benthamiana* leaves. *N. benthamiana* leaves were then inoculated 24 h later with *P. capsici*. Lesions were assessed under UV light at 48 hpi (upper panel). Lesion areas were quantified with data from 12 biologically independent samples (lower panel). Different letters represent statistically significant differences according to the Student's t-test (p-value < 0.01). P-values are shown in the Source Data. **e-f** Transient expression of BCG1 in *N. benthamiana* leaves leads to enormous H_2O_2 accumulation (**e**) and extensive callose deposition (**f**).

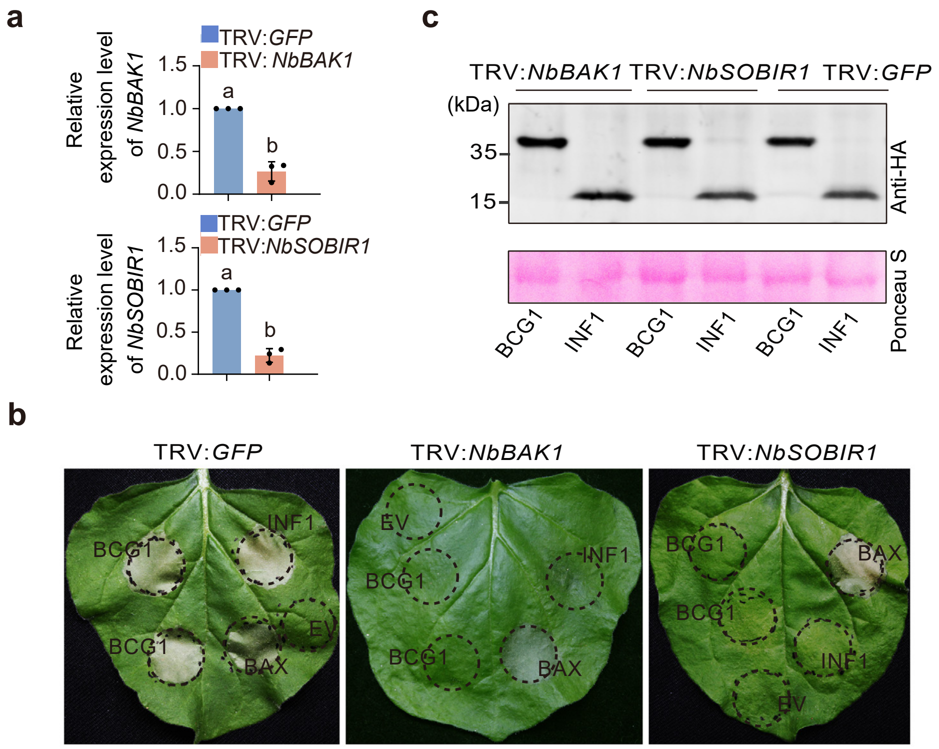

Supplementary Fig. 11. BCG1-triggered cell death requires NbBAK1 and NbSOBIR1. a *NbBAK1* and *NbSOBIR1* expression levels were determined by RT-qPCR analysis after VIGS treatment. *EF1a* was used as an internal control. The values are the means ± SD (*n*=3, biologically independent experiments). Different letters represent statistically significant differences according to the Student's *t*-test (*p*-value < 0.01). *P*-values are shown in the Source Data. **b** *N. benthamiana* leaves were subjected to VIGS inoculation with TRV:*NbBAK1*, TRV:*NbSOBIR1*, and TRV:*GFP*. After three weeks inoculation, *BCG1-*, *INF1-*, *EV-*, and *BAX-PVX* were transiently expressed in silenced *N. benthamiana* leaves and then the representative leaves were photographed at 5 dpi. **c** Western blot analysis of BCG1- and INF1-HA expression in silenced *N. benthamiana* leaves.

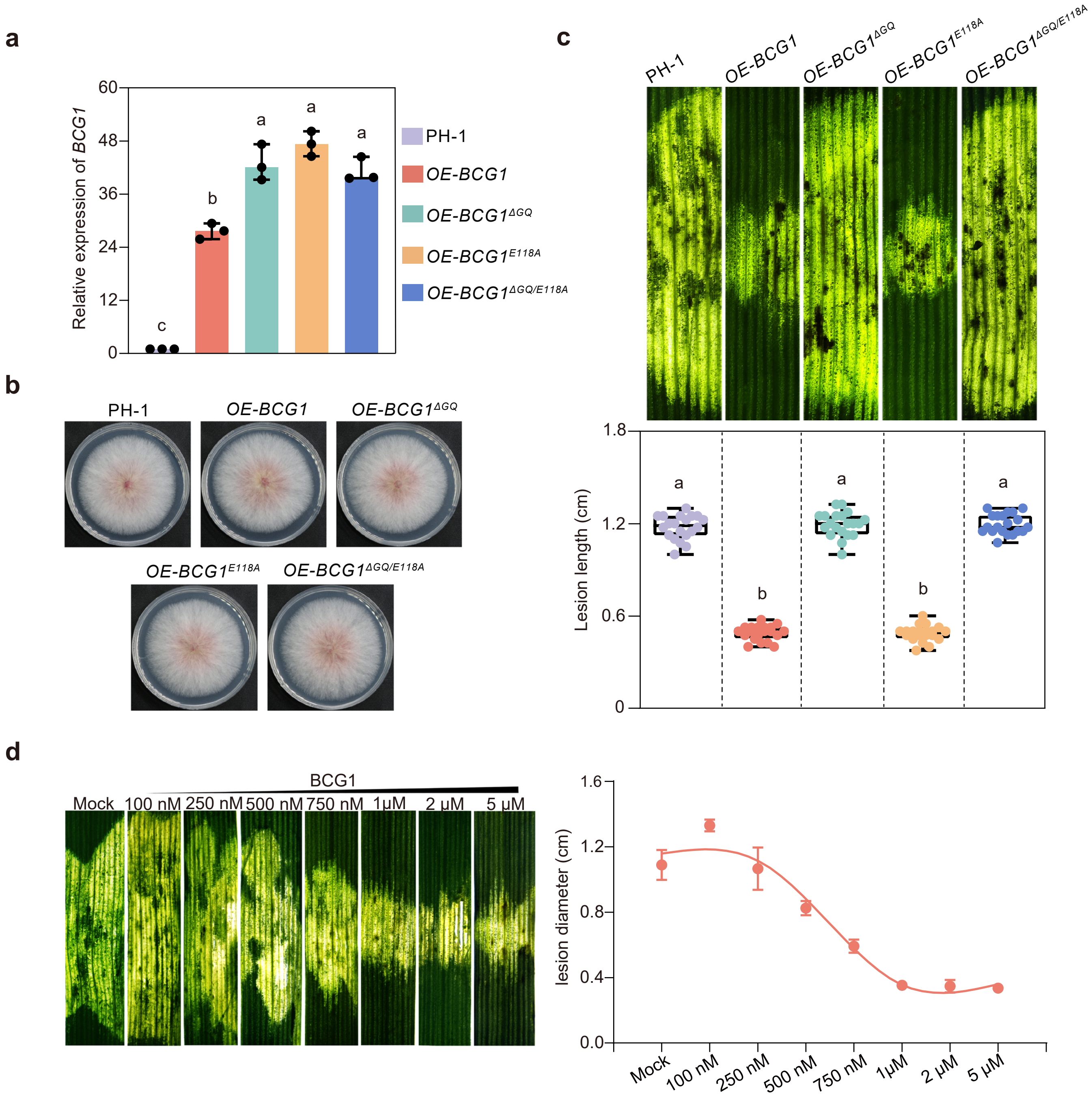

a
Relative expression of BCG1
0
12
24
36
48
60
c
b
a
a
a
PH-1
OE-BCG1
OE-BCG1ΔGQ
OE-BCG1E118A
OE-BCG1ΔGQ/E118A
b
PH-1
OE-BCG1
OE-BCG1ΔGQ
OE-BCG1E118A
OE-BCG1ΔGQ/E118A
c
PH-1
OE-BCG1
OE-BCG1ΔGQ
OE-BCG1E118A
OE-BCG1ΔGQ/E118A
Lesion length (cm)
0
0.6
1.2
1.8
a
b
a
b
a
d
BCG1
Mock
100 nM
250 nM
500 nM
750 nM
1μM
2 μM
5 μM
lesion diameter (cm)
0.0
0.4
0.8
1.2
1.6
Mock
100 nM
250 nM
500 nM
750 nM
1μM
2 μM
5 μM

Supplementary Fig. 12 BCG1 overexpression *F. graminearum* strains showed normal filamentous growth, but decreased virulence on wheat seedling leaves. a Transcript levels of *BCG1* in each overexpression strain was measured by RT-qPCR and normalized to the level in PH-1. *ACTIN* gene served as an internal reference. The values are the means ± standard deviation (*n*=3, biologically independent experiments). Different letters indicate significant differences (*p*-value < 0.01, one-way ANOVA). **b** *BCG1* overexpression strains showed normal filamentous fungal growth. *F. graminearum* wild-type strain PH-1 and the indicated overexpression strains were cultured on PDA at 25°C for 3 days. **c** Wheat seedling leaves were inoculated with the fresh mycelial plugs of the indicated *F. graminearum* strains at room temperature. Representative images of infected wheat seedling leaves were photographed at 3 days post-inoculation (dpi) (upper panel). The mean and standard deviation of the lesion length of each strain were estimated with data from 20 biologically independent samples (lower panel). Different letters represent significant differences statistically according to the one-way ANOVA followed by Fisher's LSD test (*p*-value < 0.01). *P*-values for **a**, **c** are shown in the Source Data. **d** Disease resistance of wheat seedling leaves to *F. graminearum* wild-type PH-1 triggered by indicated concentrations of BCG1 protein. Wheat seedling leaves were pretreated with purified BCG1 protein (from 100 nM to 5 μM) or the His control 24 hours before *F. graminearum* inoculation. Representative images of infected wheat seedling leaves were photographed at 3 dpi. The values are the means ± standard deviation (*n*=3, biologically independent experiments).

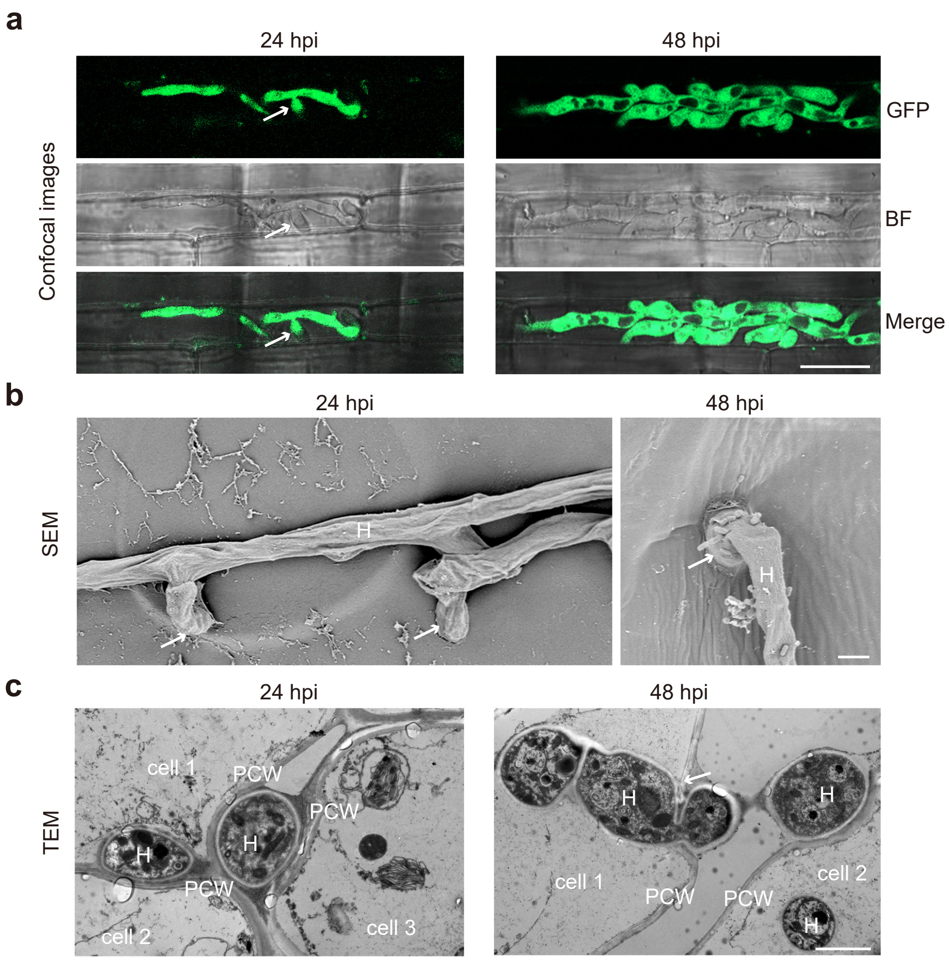

Supplementary Fig. 13 The infection process of *F. graminearum* on wheat seedling leaves. **a** Confocal images of GFP-tagged *F. graminearum* infection of wheat seedling leaves at 24 and 48 hours post-inoculation (hpi). GFP, green fluorescent protein; BF, bright field. White arrowheads indicate hyphal penetration of the wheat cell wall. White scale bars represent 20 μm. **b-c** Scanning electron microscopic (SEM) (**b**) and transmission electron microscopic (TEM) (**c**) images of wheat seedling leaves infected by *F. graminearum* wild-type PH-1 at 24 and 48 hpi. White arrowheads indicate hyphal penetration of the wheat cell wall. PCW: plant cell wall; H: *F. graminearum* hyphae. Scale bars = 2 μm.

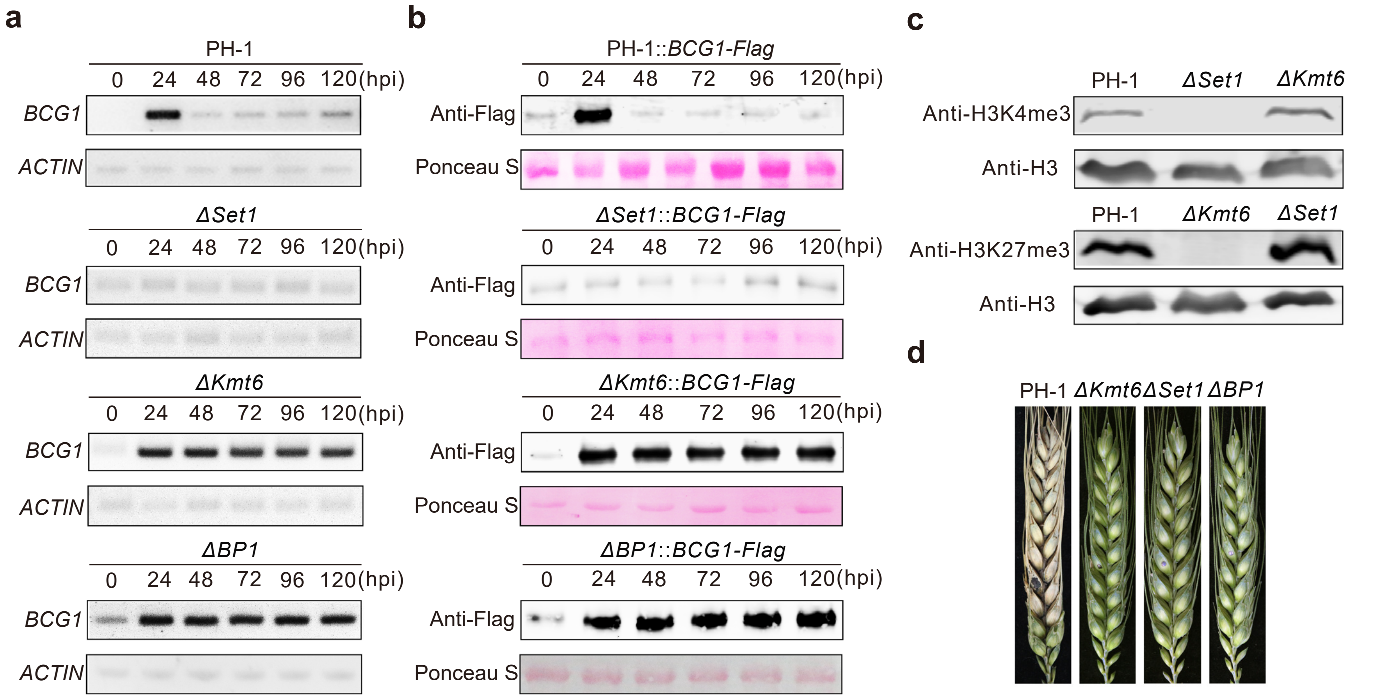

Supplementary Fig. 14 Expression dynamics of BCG1 in *ΔSet1*, *ΔKmt6* and *ΔBP1* during infection. a-b RT-PCR experiments (**a**) and western blot analysis (**b**) showing BCG1 expression in wild-type strain PH-1 and mutants (*ΔSet1*, *ΔKmt6*, and *ΔBP1*) during *F. graminearum* infection at 0-120 hours post-inoculation (hpi). Primers used for detecting *BCG1* gene expression were listed in supplementary Table 1. *ACTIN* gene was amplified as a positive control. The amounts of BCG1-Flag at 0-120 hpi were detected by western blot using an anti-Flag antibody. Protein loading is indicated by Ponceau S staining. **c** The amounts of H3K4me3 or H3K27me3 were detected by western blot using indicated antibodies. The protein samples were also detected with an anti-H3 antibody used as a loading control. **d** Virulence of PH-1 and the indicated mutants were evaluated on wheat heads. Representative images of infected wheat heads were photographed at 14 dpi.

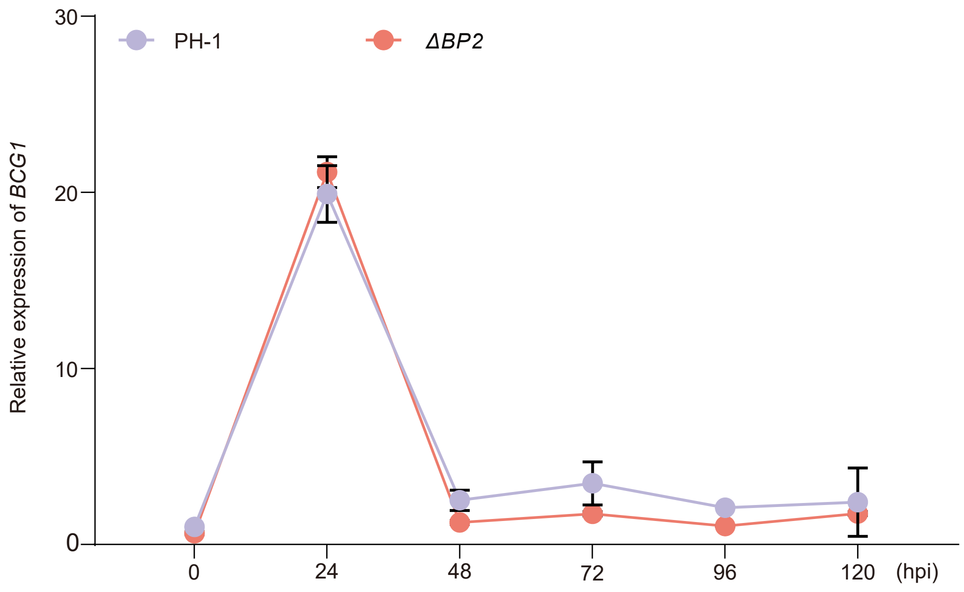

Supplementary Fig. 15 Relative expression level of *BCG1* in wild-type PH-1 and *ΔBP2* strains at 0-120 hours post-inoculation were evaluated by RT-qPCR. *ACTIN* gene was amplified as the positive control. Data presented are the means ± standard deviation (*n*=3, biologically independent experiments).

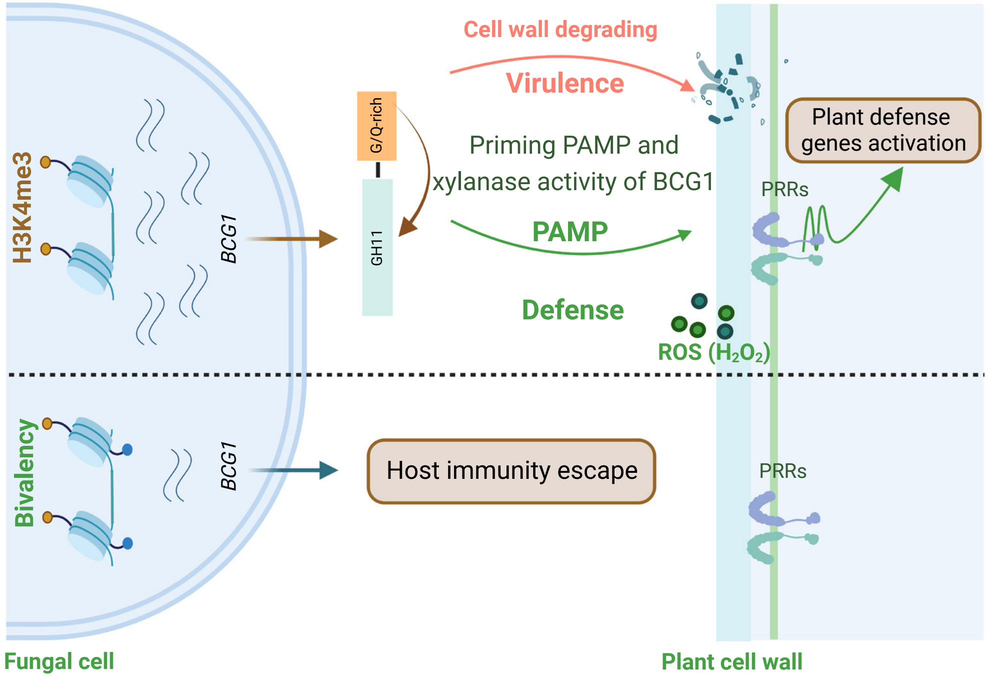

Supplementary Fig. 16 A proposed model showing bivalent histone modifications of *BCG1*-mediated host immunity escape in *F. graminearum*. Figure created using BioRender (http://biorender.com/).

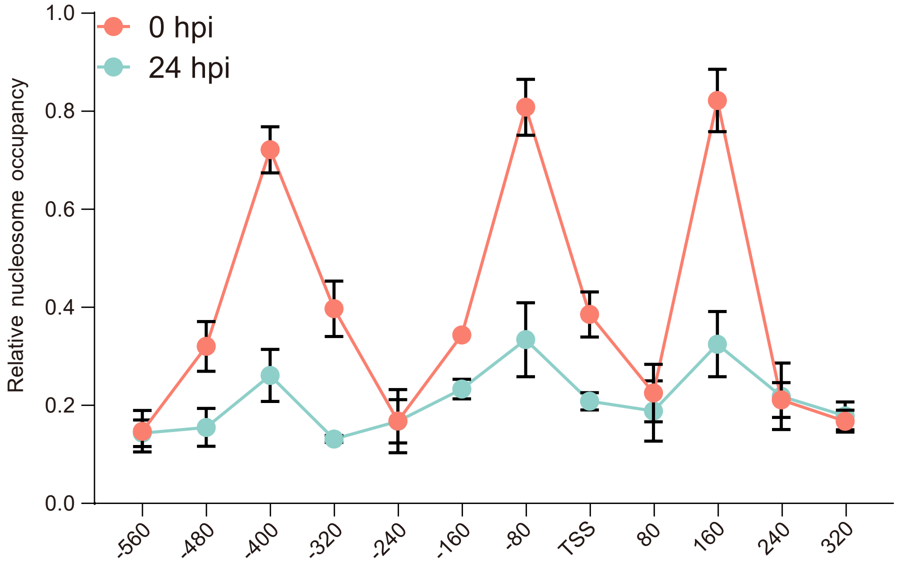

Supplementary Fig. 17 Nucleosome occupancy at *BCG1* in PH-1 at 0 and 24 hpi. Nucleosome occupancy at the genomic region of the target gene *BCG1* (-580 to 320 bp) in wild-type PH-1 as determined by MNase-qPCR. The values are the means ± standard deviation (*n*=3, biologically independent experiments).

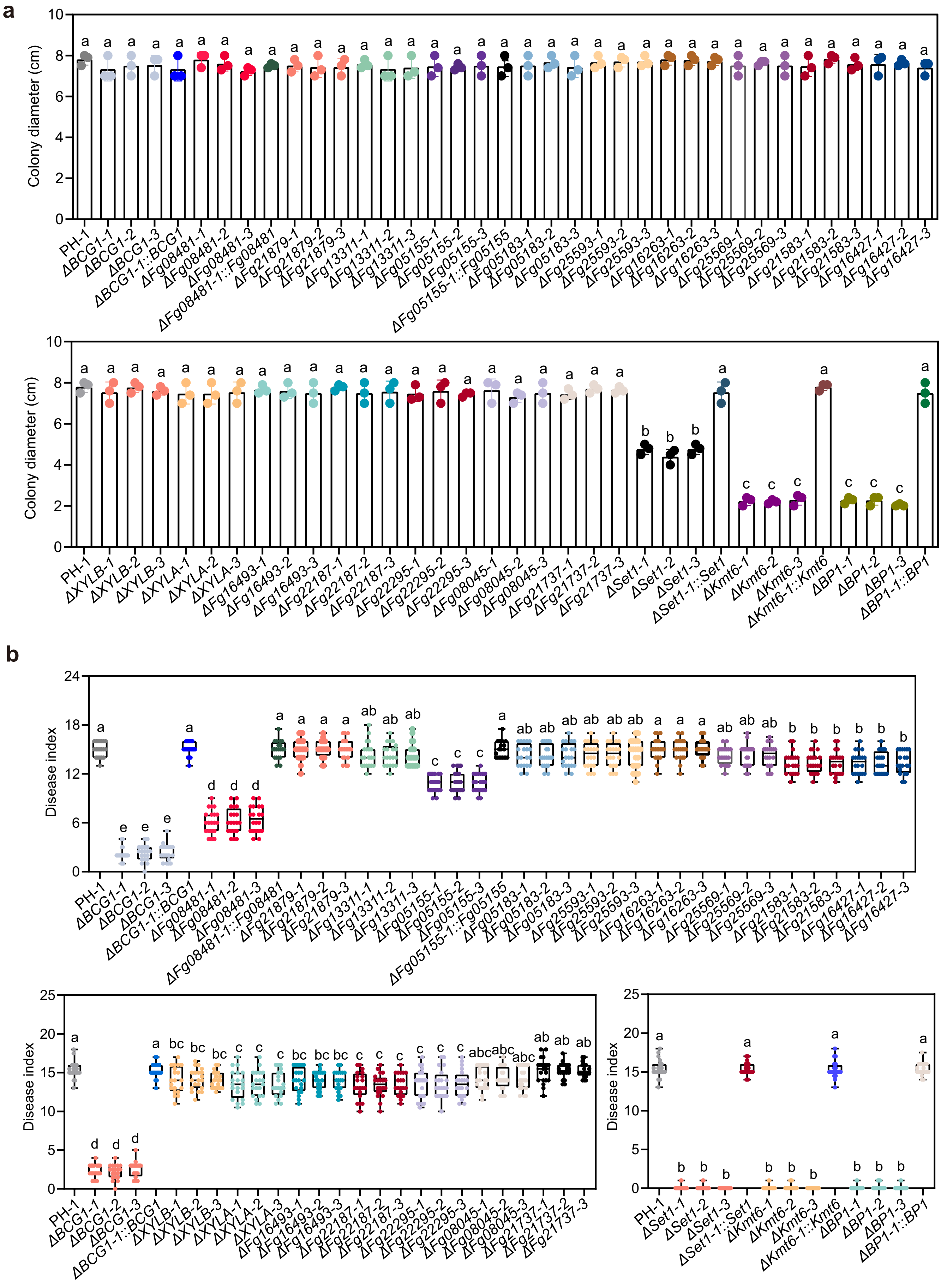

Supplementary Fig. 18 All of the knockout mutants in this study were characterized for defects in vegetative growth (a) and wheat infection (b). Different letters indicate significant differences (*p*-value < 0.01, one-way ANOVA). *P*-values are shown in the Source Data.

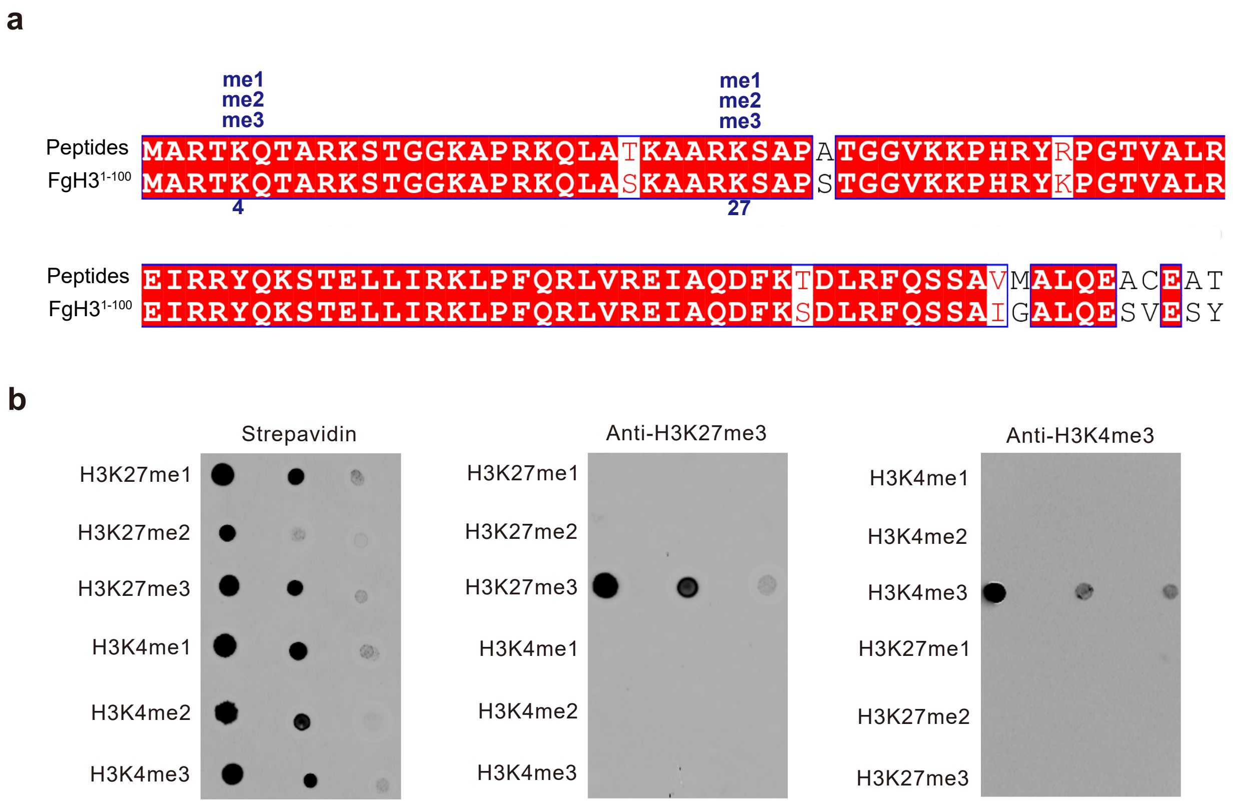

Supplementary Fig. 19 Dot blot showing the substrate specificity of the different antimodified histone antibodies. a Amino acid alignment of the indicated histone peptides with 1-100 residues of *F. graminearum* histone H3 protein (FgH3$^{1\text{-}100}$) using ClustalW, and visualizing with ESPript 3.0. **b** Different amount of biotin-labeled histone peptides (modified histone H3K4me1, H3K4me2, H3K4me3, H3K27me1, H3K27me2 and H3K27me3) were blotted to a nitrocellulose membrane. Western blot were then performed using the anti-histone antibodies indicated on the top. The Strepavidin-HRP probed membrane is shown as a control.

II. Supplementary Tables

Supplementary Table 1. Primers used in this study

Primer name	Sequence （5′-3′）
**For knockout mutants**	
BCG1-up-F1	CCAAACACCAAACACCACAT
BCG1-up-R1	CAAAATAGGCATTGATGTGTTGACCTCCATGGAAGTGATGCGAACCAT
BCG1-down-F2	CTCGTCCGAGGGCAAAGGAATAGAGTAGTTGCAAGGGCAACTAAGTTG
BCG1-down-R2	TGGTGGACTCTTTACTGTCT
BCG1-ID-F3	AAAATCGCATTTAGCCGACG
BCG1-ID-R3	CATAAGAGGTACAAAGGGCT
BCG1-up-R4	CCAAAATAGCATTGATGTGTTGACCTCCATGGAAGTGATGCGAACCAT
BCG1-down-F4	CTATCGCCTTCTTGACGAGTTCTTCTGATTGCAAGGGCAACTAAGTTG
BCG1-probe-F	GGCATGAACAATGGCTTCTT
BCG1-probe-R	AAGTGAGCACCAACATCGAT
HPH-F	GGAGGTCAACACATCAATGCCTATTTTG
HPH-R	CTACTCTATTCCTTTGCCCTCGGACGAG
HPH-probe-F	GATTCCGGAAGTGCTTGACA
HPH-probe-R	CCAGTCAATGACCGCTGTTA
Fg08481-up-F1	TTGGTAAGTCCTTGACCCATGT
Fg08481-up-R1	CAAAATAGGCATTGATGTGTTGACCTCCATGATGAGATTTCAGGCATTAGAC
Fg08481-down-F2	CTCGTCCGAGGGCAAAGGAATAGAGTAGGTGCATGGAGATGTCAAACC
Fg08481-down-R2	CTTTCTCTTCTGGGTCGTCTTC
Fg08481-ID-F3	GTTCCTACCCACTGATTATCCA
Fg08481-ID-R3	AAGTCATGTTTGAAAAGAGTACACC
Fg16263-up-F1	GACAATCACGCCGGCT
Fg16263-up-R1	CAAAATAGGCATTGATGTGTTGACCTCCGGGCTGTGTGCAGTATTG
Fg16263-down-F2	CTCGTCCGAGGGCAAAGGAATAGAGTAGGACTTTTGGCTTCGTGACTTC
Fg16263-down-R2	ATGCACAACAGTGTCAGC
Fg16263-ID-F3	ATGGGCTATGACTGGAAAGTT
Fg16263-ID-R3	CAATGTTGTCTGTGGAGCG
Fg21879-up-F1	CATGCCAATGAACAGAAAAAAC
Fg21879-up-R1	CAAAATAGGCATTGATGTGTTGACCTCCTACGGAAGTGAACCCATTATCA
Fg21879-down-F2	CTCGTCCGAGGGCAAAGGAATAGAGTAGTCATTTCGTGACTGGGTTACC
Fg21879-down-R2	GAGCTTTGGAGAGTGTGGA
Fg21879-ID-F3	TCGACTTTGTATTGCGTTCGAA
Fg21879-ID-R3	GCCTCGTTTGTATTTTTGTCGA
Fg05183-up-F1	GGTTTCTTTTATCACTTTTAGCCTT
Fg05183-up-R1	CAAAATAGGCATTGATGTGTTGACCTCCCCTGCCATATGGCATCG
Fg05183-down-F2	CTCGTCCGAGGGCAAAGGAATAGAGTAGGAGATCTATGACTCTCATCTTGACA
Fg05183-down-R2	TCCAAAGTTTTTGGGTAGCCC
Fg05183-ID-F3	AGCATCATGCAGCCGTA
Fg05183-ID-R3	GTGCAGGGAATGATCTTGGT
Fg25569-up-F1	AACTTGAATTGCCATGAGCC
Fg25569-up-R1	CAAAATAGGCATTGATGTGTTGACCTCCGTGTATGTATTGGCTGCTTTATCC
Fg25569-down-F2	CTCGTCCGAGGGCAAAGGAATAGAGTAGTATCAATGAGCGATATACGATATATT
Fg25569-down-R2	TCGAAGTCTTGCTCGAGTTT
Fg25569-ID-F3	TATTTGACACTTGGTCGGCC
Fg25569-ID-R3	GAACACGCGGTCGTTG
Fg25593-up-F1	ACCGAAGCACAATCGATG
Fg25593-up-R1	CAAAATAGGCATTGATGTGTTGACCTCCAAGGGATCAGCCTTCCAG
Fg25593-down-F2	CTCGTCCGAGGGCAAAGGAATAGAGTAGTGGACCTTTACTTGGATGAGGA
Fg25593-down-R2	AGCCAACATGAAACTTTGCC
Fg25593-ID-F3	CTTGTTCCATTTTCCATTTCGC
Fg25593-ID-R3	CAGTAATGTCGTTTACGCTG
Fg13311-up-F1	AAAGTCATTGTCGAGCTGG
Fg13311-up-R1	CAAAATAGGCATTGATGTGTTGACCTCCGAATCAAGCGACGTTTTGAGG
Fg13311-down-F2	CTCGTCCGAGGGCAAAGGAATAGAGTAGGCAGTACTGGCAAGAGCT
Fg13311-down-R2	ACTTGGTAACAATCTTGTTGACC

Fg13311-ID-F3	GCAATAGCTTCATGTAAGAGCC
Fg13311-ID-R3	GAGAATCTTGTCGTTGCTCTC
Fg16427-up-F1	TGCCGCCGTTCGACTTT
Fg16427-up-R1	CAAAATAGGCATTGATGTGTTGACCTCCTTGTGTGATTTTTCTGACGCAA
Fg16427-down-F2	CTCGTCCGAGGGCAAAGGAATAGAGTAGGCCAGTGAGACAACTAGGT
Fg16427-down-R2	ACACTCGTCCAAAGCGTC
Fg16427-ID-F3	GAAGGGAAATTTTTTCCATTGCG
Fg16427-ID-R3	AACGACTTTCCACGGCG
Fg21583-up-F1	CGCGTTACATGGGAGAGCTA
Fg21583-up-R1	CAAAATAGGCATTGATGTGTTGACCTCCATGTCAGTGGCTGATGCT
Fg21583-down-F2	CTCGTCCGAGGGCAAAGGAATAGAGTAGTGCCATCAGTAACACCAGAT
Fg21583-down-R2	CGAGCCTCAGAGAGTAAATCA
Fg21583-ID-F3	GCACTCTTATGGATCTAATTGCT
Fg21583-ID-R3	TCAAAAGGTCCGAAATGCTGG
Fg05155-up-F1	ATCGAATTTTGGGTCGATGTT
Fg05155-up-R1	CAAAATAGGCATTGATGTGTTGACCTCCCCTTTGACAAAGCATGACTGG
Fg05155-down-F2	CTCGTCCGAGGGCAAAGGAATAGAGTAGATATTTCAAGACAAGGATGACCT
Fg05155-down-R2	GCTTTCCTCATCAAAGTCTGT
Fg05155-ID-F3	CGTTTATCTTCTATGGGAGACG
Fg05155-ID-R3	ATCTCTGTTTTCCGCAGCA
XYLB-up-F1	ACGGGTTCTCTATCATGAGC
XYLB-up-R1	CCAAAATAGCATTGATGTGTTGACCTCCTTTGACTGTGATGCGCTT
XYLB-down-F2	CTATCGCCTTCTTGACGAGTTCTTCTGAACCAGATCCTTGCTGTTGAG
XYLB-down-R2	GCAGAAGCCAATCATGTCAT
XYLB-ID-F3	AATTCACACGTCGATCCTCT
XYLB-ID-R3	GCCTTGGTCAAAGGTCCTTC
Neo-F	GGAGGTCAACACATCAATGCT
Neo-R	TCAGAAGAACTCGTCAAGAAG
XYLA-up-F1	GGAGTATGGCTAGCCTACGA
XYLA-up-R1	CAAAAAATGCTCCTTCAATATCACTAGTGATGGTGAAGGGCGCGA
XYLA-down-F2	CCTAACCCTAAACCAAAGCATCAGGCCTGACGGTGTTCAGACCTTCAA
XYLA-down-R2	GGTTTGACTTGGTCTCGACA
XYLA-ID-F3	GGCTAGCCTACGATATGTGC
XYLA-ID-R3	AATGGGAAGCGAATTCGCAA
Nat1-F	ACTAGTGATATTGAAGGAGC
Nat1-R	AGGCCTGATGCTTTGGTTTAG
Fg16493-up-F1	TGTACCTGTTCCGTTGACCT
Fg16493-up-R1	CAAAATAGGCATTGATGTGTTGACCTCCCGACGAGCGCTACAATAAGG
Fg16493-down-F2	CTCGTCCGAGGGCAAAGGAATAGAGTAGTGTCCAGCGATCTTGATCTT
Fg16493-down-R2	CCATTCTGAAGGCATGTTGA
Fg16493-ID-F3	AAGTCTAAACGACAACATTGC
Fg16493-ID-R3	TGCCTTTTTATGTCCTCACAG
Fg21737-up-F1	TCAGTTGCTATCCGGGGTAG
Fg21737-up-R1	CAAAATAGGCATTGATGTGTTGACCTCCCGAGTCCTAGGAAGTGCATG
Fg21737-down-F2	CTCGTCCGAGGGCAAAGGAATAGAGTAGCAGGCTCATCTTGCCAAG
Fg21737-down-R2	AGTTGACGCTTGTTGTTTGA
Fg21737-ID-F3	GGGGGACAGTTCAGATTCAG
Fg21737-ID-R3	TCTCGCTCATCCTAATCTCC
Fg08045-up-F1	ATCACAACATGTGCAGCATT
Fg08045-up-R1	CAAAATAGGCATTGATGTGTTGACCTCCGAGGGCAGATTTGTGCATTT
Fg08045-down-F2	CTCGTCCGAGGGCAAAGGAATAGAGTAGGTGCATTGAGGCTTAAGTGA
Fg08045-down-R2	GCTGGAAGGATTGAAGGTGA
Fg08045-ID-F3	GGACTTGGGACGATGAACAT
Fg08045-ID-R3	TCCTCAGGTTTGCTTTGGAG
Fg22295-up-F1	AACACAGTGATTTAGCCGGG
Fg22295-up-R1	CAAAATAGGCATTGATGTGTTGACCTCCAACCAGGCCATTGTAAAGGG
Fg22295-down-F2	CTCGTCCGAGGGCAAAGGAATAGAGTAGTGGTGAGCATCTCGTAGAGT
Fg22295-down-R2	CATCCAAGTTTGGGAAGGGC

Fg22295-ID-F3	GGCCTTGGCCCTTTACA
Fg22295-ID-R3	TTACTCTACGAGATGCTCAC
Fg22187-up-F1	GTCGTCTCACTCTCATCTCA
Fg22187-up-R1	CAAAATAGGCATTGATGTGTTGACCTCCAAAGAGGAGGGAAGAGAACT
Fg22187-down-F2	CTCGTCCGAGGGCAAAGGAATAGAGTAGTGTCAACGCTCTCCGTTAAG
Fg22187-down-R2	CGATGGTTTTCCCTCAGGAC
Fg22187-ID-F3	GCACTTGGCATCCATCAC
Fg22187-ID-R3	TGAAGCCCCCTGTGCTTC
FgSet1-up-F1	ACCATCGACTCCACAACAGGT
FgSet1-up-R1	CAAAATAGGCATTGATGTGTTGACCTCCACGAGCTGCATCAATACCAGA
FgSet1-down-F2	CTCGTCCGAGGGCAAAGGAATAGAGTAGCCTGCTCTTGAGTTACCGGA
FgSet1-down-R2	TGCCGCAGAGACAAGGAATA
FgSet1-ID-F3	TCGTGACAAGATACAATCCGC
FgSet1-ID-R3	AACGTGCGTCTTGCAGAGTT
FgKMT6-up-F1	CAGGACCAATCGAAGGAC
FgKMT6-up-R1	CAAAATAGGCATTGATGTGTTGACCTCCGGCTGCGGCTAATTGATG
FgKMT6-down-F2	CTCGTCCGAGGGCAAAGGAATAGAGTAGATCTGGGTGGACGCTGCCACG
FgKMT6-down-R2	GCGCTCATCCAATCTCCATC
FgKMT6-ID-F3	GGTGGTAGTTGCGAGCCAAA
FgKMT6-ID-R3	GGCGTGGTTGATGTAACG
FgBP1-up-F1	GGCCGACAATTGTGGGCG
FgBP1-up-R1	CAAAATAGGCATTGATGTGTTGACCTCCGTCTTTGAGCGACAGATCG
FgBP1-down-F2	CTCGTCCGAGGGCAAAGGAATAGAGTAGCAGTTTCTTTTGTCAAAATT
FgBP1-down-R2	GCGATAGAGATAATAGCGTT
FgBP1-ID-F3	GGCTCCCCTGTATGCTTCTA
FgBP1-ID-R3	GCGGTGGGAAACAAATACCA
Fg06833-up-F1	TTACCTAGCCACCACCACA
Fg06833-up-R1	CAAAATAGGCATTGATGTGTTGACCTCCGACATGCAGCAGATAATCAC
Fg06833-down-F2	CTCGTCCGAGGGCAAAGGAATAGAGTAGCTTAACGAACGGTCAATGGC
Fg06833-down-R2	CAATTGACTGTATGACGACA
Fg06833-ID-F3	GCTCTAAAACAACCCCTGCC
Fg06833-ID-R3	ACCATGGAAACGAGAAATCAGG
**For complementation mutants**	
BCG1-C-F1	ACTCACTATAGGGCGAATTGGGTACTCAAATTGGTTATCCCATGGATGTTTGCC
BCG1-C-R2	CACCACCCCGGTGAACAGCTCCTCGCCCTTGCTCACGTTGCCCTTGCAAGACTCT
BCG1-E118A-C-R1	CGAAATCCTCGACGATGTAGTA CGCGATCAAGGGGTTT
BCG1-E118A-C-F2	TACTACATCGTCGAGGATTTCG
BCG1-E209A-C-R1	GATCCGCTGCTCAAGTAGCC CGCAGTGGCGAGGAT
BCG1-E209A-C-F2	GGCTACTTGAGCAGCGGATC
BCG1Δ^{GQ}-C-R2	CACCACCCCGGTGAACAGCTCCTCGCCCTTGCTCACGGAGACGGTCATGTGGGAA
BCG1-C-ID-F	ATCCCATGGATGTTTGCC
BCG1-C-ID-R	TCTTCAGCAGCCTGCTGC
Fg08481-C-F	ACTCACTATAGGGCGAATTGGGTACTCAAATTGGTTGAGAGATTTTGGGAACGGTC
Fg08481-C-R	CACCACCCCGGTGAACAGCTCCTCGCCCTTGCTCACCTTACCCCATTTCTCTATTCTAGC
Fg08481-C-ID-F	AAGATGCATAGGCTTGTTCCT
Fg08481-C-ID-R	CTGTCAAACCAAACAGCTCT
Fg05155-C-F	ACTCACTATAGGGCGAATTGGGTACTCAAATTGGTTAAGTAAACACAGCCGCAATAG
Fg05155-C-R	CACCACCCCGGTGAACAGCTCCTCGCCCTTGCTCACTATGACGTATATGGAATGAACACC
Fg05155-C-ID-F	TCGATGAGGCCCGAAATTAT
Fg05155-C-ID-R	AGGCTCTTTTCTTTATCCGT
FgKmt6-C-F	ACTCACTATAGGGCGAATTGGGTACTCAAATTGGTTATCCCGACGCTCACGTGAGA
FgKmt6-C-R	CACCACCCCGGTGAACAGCTCCTCGCCCTTGCTCACCTGACTGGCGCTCATCCAAT
FgKmt6-C-ID-F	ATCCCGACGCTCACGTGAGA
FgKmt6-C-ID-R	TCTTCATTCCAGGGTCGC
FgSet1-C-F	ACTCACTATAGGGCGAATTGGGTACTCAAATTGGTTAAAGATACCCTGCTTTAGACGT
FgSet1-C-R	CACCACCCCGGTGAACAGCTCCTCGCCCTTGCTCACGTTGAGGAAGCCCTTGCAG
FgSet1-C-ID-F	AAAGATACCCTGCTTTAGACGT
FgSet1-C-ID-R	GTGCCAAGCAAGGGTATT

FgBP1-C-F	ACTCACTATAGGGCGAATTGGGTACTCAAATTGGTTGAAAGTGACGATGGAGCTGA
FgBP1-C-R	CACCACCCCGGTGAACAGCTCCTCGCCCTTGCTCACTTCAATACAGTTGCCACATATCAG
FgBP1-C-ID-F	GAAAGTGACGATGGAGCTGA
FgBP1-C-ID-R	ACGGCGTTCCATCAGATAAG
**For overexpression**	
gpda-F:	CACCTTCAGTGGACTCGAG
gpda-R:	TGTGATGTCTGCTCAAGCGG
OE-BCG1-F	CCGCTTGAGCAGACATCACAATGGTTCGCATCACTTCCAT
OE-BCG1-R	CAAAATAGGCATTGATGTGTTGACCTCCTTAGTTGCCCTTGCAAGACT
OE-BCG1Δ^{GQ} -R	CAAAATAGGCATTGATGTGTTGACCTCCTTAGGAGACGGTCATGTGGGAA
**For ChIP-qPCR、ReChIP-qPCR**	
BCG1-P1-F	TGTTCTGCGAACATATCTGT
BCG1-P1-R	CTCGCTCTATCTGTTGGTAT
BCG1-P2-F	CACTTATGAGAACGGCGC
BCG1-P2-R	GTAAACGGCATTGGAGTATG
BCG1-P3-F	ATGGTTCGCATCACTTCC
BCG1-P3-R	TTCATGCCCGAGCTGTTG
BCG1-P4-F	ATACCACAGAGTACACACCA
BCG1-P4-R	GAACATGAATCGATCGTGGG
FgKmt6GFP-F	TTTCGTAGGAACCCAATCTTCAAAATGGCTTCACAATCTGTCAT
FgKmt6GFP-R	CACCACCCCGGTGAACAGCTCCTCGCCCTTGCTCACCTGACTGGCGCTCATCCA
FgSet1GFP-F	TTTCGTAGGAACCCAATCTTCAAAATGACTCGCCCGCCAG
FgSet1GFP-R	CACCACCCCGGTGAACAGCTCCTCGCCCTTGCTCACGTTGAGGAAGCCCTTGCA
FgBP1GFP-F	TTTCGTAGGAACCCAATCTTCAAAATGAGCAATCGCAAACGATC
FgBP1GFP-R	CACCACCCCGGTGAACAGCTCCTCGCCCTTGCTCACTTCAATACAGTTGCCACATATCAG
**For protein expression**	
BCG1-F	CCGGAATTCGCCCCCTCTCCTGAAGGCATGTT
BCG1-R	AAGGAAAAAAGCGGCCGCGTTGCCCTTGCAAGACTCTT
BCG1Δ^{GQ}-R	AAGGAAAAAAGCGGCCGCGGAGACGGTCATGTGGGAA
XYLB-F	CCGGAATTCGCTCCCAACCCTACCAAG
XYLB-R	AAGGAAAAAAGCGGCCGCTCCAGAGACAGTCATGGTAGC
XYLA-F	CCGGAATTC CGTCCCTTTGACTTCCTCGA
XYLA-R	AAGGAAAAAAGCGGCCGCACTAGTCTGGACATAGATAGAA
FvBCG1-F	CCGGAATTC GCGCCCTCCAAAGAAGGT
FvBCG1-R	AAGGAAAAAAGCGGCCGCTTGGCAGGGCTCGTTAG
Fv BCG1Δ^{GQ} -R	AAGGAAAAAAGCGGCCGCAGAAACGGTCATGTGAGAAG
FoBCG1-F	CCGGAATTC GCGCCCTCCAAAGAAGGT
FoBCG1-R	AAGGAAAAAAGCGGCCGCATCTCTTGCTGCTGGGGT
FoBCG1Δ^{GQ}-R	AAGGAAAAAAGCGGCCGCAGAGACGGTCATGTGGGA
**For transient expression**	
pGR107-BCG1-F	TCCCCCGGGATGGTTCGCATCACTTCCAT
pGR107-BCG1-R	AAGGAAAAAAGCGGCCGCGTTGCCCTTGCAAGACTCTT
pGR107-BCG1Δ^{SP}-F	TCCCCCGGGGCCCCCTCTCCTGAAGGCATGTT
pGR107-PR1-BCG1-F1	TCCCCCGGGATGGGATTTGTTCTCTTTTCGCA
pGR107-PR1-BCG1-R1	AACATGCCTTCAGGAGAGGGGGCTGACCTGGCACGGCAAGAGT
pGR107-PR1-BCG1-F2	GCCCCCTCTCCTGAAGGCATGTT
pGR107- BCG1Δ^{GQ} -R	AAGGAAAAAAGCGGCCGCGGAGACGGTCATGTGGGAA
pGR107-BCG1E118A-R1	CGAAATCCTCGACGATGTAGTACGCGATCAAGGGGTTT
pGR107-BCG1E118A-F2	TACTACATCGTCGAGGATTTCG
pGR107-G/Q-F	TCCCCCGGGGAGGGTGCTTCTTCCGCT
pBinGFP-BCG1-F	TCCCCCGGGATGGTTCGCATCACTTCCAT
pBinGFP-BCG1-R	CGCGGATCC GTTGCCCTTGCAAGACTCTT
pBinGFP-BCG1Δ^{GQ}-F	CGCGGATCCGGAGACGGTCATGTGGGAA
**For RT-qPCR**	
NbSOBIR1-RT-F	CTTAGAAAAACTCTCTTTAGC
NbSOBIR1-RT-R	TATGGATTGGAGTGACATTATG
NbBAK1-RT-F	ATTGCTGGAGGAGTTGCTGCCGG
NbBAK1-RT-R	CCACGTACAGCAGTGGTAACAT

NbEF1α-RT-F	GTATGCCTGGGTGCTTGAC
NbEF1α-RT-R	ACAGGGACAGTTCCAATACCA
RT-BCG1-F	ATGGTTCGCATCACTTCC
RT-BCG1-R	TTCATGCCCGAGCTGTTG
RT-FgACTIN-F	GGAGATCACTGCTCTTGCTC
RT-FgACTIN-R	CTGCTTGGAGATCCACATTT
RT-AtPR1-F	TTCTTCCCTCGAAAGCTCAA
RT-AtPR1-R	AAGGCCCACCAGAGTGTATG
RT-FRK1-F	GGAAGCGGTCAGATTTCAAC
RT-FRK1-R	AGCTTGCAATAGCAGGTTGG
RT-AtACTIN-F	ATGGAAGCTGCTGGAATCCAC
RT-AtACTIN-R	TTGCTCATACGGTCAGCGATA
RT-TaPR1-F	GAGAATGCAGACGCCCAAGC
RT-TaPR1-R	CTGGAGCTTGCAGTCGTTGATC
RT-TaERF113-F	GAACAGAGGGAGACCTTG
RT-TaERF113-R	TTCAATTTTGCAAACAATGT
RT-TaWRKY28-F	GGAGAGGACGATGCTTGC
RT-TaWRKY28-R	CATCAGCGCAGCAGGTAC
RT-TaGAPDH-F	TTAGACTTGCGAAGCCAGCA
RT-TaGAPDH-R	AAATGCCCTTGAGGTTTCCC
**For flag**	
BCG1-flag-F	AAGTGTGTCTTGATGCTCGAGATCCCATGGATGTTTGCC
BCG1-flag-R	ATGGTCTTTGTAGTCCTCGAGGTTGCCCTTGCAAGACTC
ACTIN-flag-F	AAGTGTGTCTTGATGCTCGAGATGGAGGAAGAAGTTGCT
ACTIN-flag-R	ATGGTCTTTGTAGTCCTCGAGGAAGCACTTGCGGTGAACGA
**MNase-qPCR**	
ACTIN-F	ATGGAGGGTACGTCGCATCATA
ACTIN-R	GTCAGCGTGAATCCTCAG
BCG1-F1	CAAACACCACATAGTTGTAT
BCG1-R1	GTATTTTAGTATTCGAATGA
BCG1-F2	TCATTCGAATACTAAAATAC
BCG1-R2	AAGGCGATGTTTATGCTGTC
BCG1-F3	GACAGCATAAACATCGCCTT
BCG1-R3	TATGAAACCGCAAAATCAAT
BCG1-F4	ATTGATTTTGCGGTTTCATA
BCG1-R4	TCGGACCATGTTAATGAAGG
BCG1-F5	CCTTCATTAACATGGTCCGA
BCG1-R5	GATACTCAAGAATCAAAAGA
BCG1-F6	TCTTTTGATTCTTGAGTATC
BCG1-R6	GGTGAGGTGAAAGGATGATC
BCG1-F7	GATCATCCTTTCACCTCACC
BCG1-R7	TTTGGCGAAAGATTTTTGAT
BCG1-F8	ATCAAAAATCTTTCGCCAAA
BCG1-R8	AACATGCCTTCAGGAGAGGG
BCG1-F9	CCCTCTCCTGAAGGCATGTT
BCG1-R9	CATCAGACCACCAAGAGTAG
BCG1-F10	CTACTCTTGGTGGTCTGATG
BCG1-R10	ACCGACGACGTTGCCACCAG
BCG1-F11	CTGGTGGCAACGTCGTCGGT
BCG1-R11	GTTCTGGTGATGTTAGCGTG
BCG1-F12	CACGCTAACATCACCAGAAC
BCG1-R12	CGATCAAGGGGTTTCGGGTC

Supplement Table 2. Parameters of softs for RNA-Seq, ChIP-Seq and sequential ChIP-seq data analysis

For RNA-Seq data analysis:

Softs	Parameters
fastp v0.18.0	fastp --in1 READ1-FASTQ-FILE --out1 READ1-OUT-FILE --in2 READ2-FASTQ-FILE --out2 READ2-OUT-FILE -q 20
hisat2 v1.3.3	hisat2 -p 56 -x index of reference genome -1 READ1-INPUT-FILE -2 READ2-INPUT-FILE -t -q -S --phred33 --summary
samtools v1.9	samtools sort SAM-FLIE -O bam -@ 16 -o OUT-FILE
stringtie v1.2.0	stringtie INPUT-BAM-FILE -G genome annotation file -v -e -o OUT-FILE

For ChIP-Seq and sequential ChIP-seq data analysis:

Softs	Parameters
SOAPnuke v2.1.7	SOAPnuke -1 FASTQ-FILE -C OUT-FIL -l 15 -q 0.4 -n 0.01
bowtie2 v2.4.5	bowtie2 -x index of reference genome -U INPUT-FILE -q -t -p 56 -U --phred33 -S OUT-FILE
samtools v1.9	samtools sort SAM-FLIE -O bam -@ 16 -o OUT-FILE
deeptools v2.4.1	bamCompare -b1 IP-BAN-FILE -b2 INPUT-BAM-FILE -o OUT-BW-FILE --scaleFactorsMethod None --operation log2 --normalizeUsing BPM -p 56
