# Supplementary material for: Temporally-coordinated bivalent histone modifications of BCG1 enable fungal invasion and immune evasion
Source: Nat Commun. 2024 Jan 5;15:231. doi: 10.1038/s41467-023-44491-6 (PMC10770383; doi:10.1038/s41467-023-44491-6)
Supplement: Supplementary file 3 — Reporting Summary [file 41467_2023_44491_MOESM3_ESM.pdf]

nature research

Corresponding author(s): Qin Gu

Last updated by author(s): Dec 3, 2023

Reporting Summary

Nature Research wishes to improve the reproducibility of the work that we publish. This form provides structure for consistency and transparency in reporting. For further information on Nature Research policies, see our Editorial Policies and the Editorial Policy Checklist.

Statistics

For all statistical analyses, confirm that the following items are present in the figure legend, table legend, main text, or Methods section.

n/a	Confirmed	
☐	☒	The exact sample size (*n*) for each experimental group/condition, given as a discrete number and unit of measurement
☐	☒	A statement on whether measurements were taken from distinct samples or whether the same sample was measured repeatedly
☐	☒	The statistical test(s) used AND whether they are one- or two-sided<br>*Only common tests should be described solely by name; describe more complex techniques in the Methods section.*
☒	☐	A description of all covariates tested
☒	☐	A description of any assumptions or corrections, such as tests of normality and adjustment for multiple comparisons
☐	☒	A full description of the statistical parameters including central tendency (e.g. means) or other basic estimates (e.g. regression coefficient) AND variation (e.g. standard deviation) or associated estimates of uncertainty (e.g. confidence intervals)
☐	☒	For null hypothesis testing, the test statistic (e.g. *F*, *t*, *r*) with confidence intervals, effect sizes, degrees of freedom and *P* value noted<br>*Give P values as exact values whenever suitable.*
☒	☐	For Bayesian analysis, information on the choice of priors and Markov chain Monte Carlo settings
☒	☐	For hierarchical and complex designs, identification of the appropriate level for tests and full reporting of outcomes
☒	☐	Estimates of effect sizes (e.g. Cohen's *d*, Pearson's *r*), indicating how they were calculated

Our web collection on statistics for biologists contains articles on many of the points above.

Software and code

Policy information about availability of computer code

Data collection	The RNA-seq data were collected by Illumina HiSeq4000.The ChIP-seq data were collected by Illumina HiSeq4000.
Data analysis	For RNA-seq data analysis, all of the raw reads were filtered by Fastp (version 0.18.0) software. Clean reads were aligned to the reference genome of F. graminearum strain PH-1(accession number: GCA_900044135.1) or wheat Triticum aestivum cv. Chinese Spring (IWGSC RefSeq v2.1) using Hisat2 (version 1.3.3).Genes displaying log2[fold change] ≥ 1.5 or ≤ -1.5 and an adjusted p-value < 0.05 were classified as differentially expressed genes (DEGs) by DESeq2 package. Expression levels for mRNA in each sample were quantified to TPM (Transcripts Per Million mapped reads) using StringTie (version 1.2.0). For GO analysis in wheat plants, the top21 categories termed “biological process” with a q-value less than 0.05 were chosen for visualization.<br>For ChIP-seq and sequential ChIP-seq data analysis, raw reads were were filtered using SOAPnuke (version 2.1.7). Clean reads were mapped to the reference genome of F. graminearum strain PH-1(accession number: GCA_900044135.1) by Bowtie2 (version 2.4.5) with default parameters. BPM (Bins Per Million mapped reads) were calculated to normalized reads using bamCompare tool in Deeptools (version 2.4.1) with input DNA as a control. The resulting bigwig files were then loaded into genome browser IGV (version 2.8.9) and visually analyzed. MACS2 (version 2.1.4) was used to call peaks. The peaks were annotated using R package ChIPseeker (version 1.26.2). R package DiffBind (version 3.0.15) was used to identify differentially enriched ChIP peaks between 48 hpi and 0 hpi.

For manuscripts utilizing custom algorithms or software that are central to the research but not yet described in published literature, software must be made available to editors and reviewers. We strongly encourage code deposition in a community repository (e.g. GitHub). See the Nature Research guidelines for submitting code & software for further information.

Data

Policy information about availability of data

All manuscripts must include a data availability statement. This statement should provide the following information, where applicable:

- Accession codes, unique identifiers, or web links for publicly available datasets
- A list of figures that have associated raw data
- A description of any restrictions on data availability

RNA-seq and ChIP-seq data were deposited in the NCBI BioProject database with accession code GSE213962 (https://www.ncbi.nlm.nih.gov/geo/query/acc.cgi?acc=GSE213962). Other relevant data supporting the findings of the study are available in this manuscript and the Supplementary Information files.

Field-specific reporting

Please select the one below that is the best fit for your research. If you are not sure, read the appropriate sections before making your selection.

[x] Life sciences [ ] Behavioural & social sciences [ ] Ecological, evolutionary & environmental sciences

For a reference copy of the document with all sections, see nature.com/documents/nr-reporting-summary-flat.pdf

Life sciences study design

All studies must disclose on these points even when the disclosure is negative.

Sample size	No statistical methods were used to determine sample size. Detailed sample size for each experiment can be found in the methods and Figure legend part.
Data exclusions	No data were excluded
Replication	All attempts at replication were successful
Randomization	Experiment materials were collected randomly without bias.
Blinding	N/A

Reporting for specific materials, systems and methods

We require information from authors about some types of materials, experimental systems and methods used in many studies. Here, indicate whether each material, system or method listed is relevant to your study. If you are not sure if a list item applies to your research, read the appropriate section before selecting a response.

Materials & experimental systems

n/a	Involved in the study	
[ ]	[x]	Antibodies
[x]	[ ]	Eukaryotic cell lines
[x]	[ ]	Palaeontology and archaeology
[x]	[ ]	Animals and other organisms
[x]	[ ]	Human research participants
[x]	[ ]	Clinical data
[x]	[ ]	Dual use research of concern

Methods

n/a	Involved in the study	
[ ]	[x]	ChIP-seq
[x]	[ ]	Flow cytometry
[x]	[ ]	MRI-based neuroimaging

Antibodies

Antibodies used	For Western Blot, primary antibodies include anti-HA antibody (M20003M, Abmart, Shanghai, China); anti-FLAG antibody (A9044, Sigma, St. Louis, MO); anti-GFP antibody (ab32146, Abcam, Cambridge, UK); anti-His antibody (ab18184, Abcam, Cambridge, MA, USA); anti-H3K27me3 antibody (Thermofisher, 39155, MA, USA); anti-H3K4me3 antibody (Abcam, ab8580, Cambridge, UK); anti-H3 antibody (ab1791, Abcam, Cambridge, UK) . Secondary antibodies include anti-Mouse antibody (926-32210, LI-COR, Lincoln, NE, USA); anti-Rabbit antibody (926-32211, LI-COR, Lincoln, NE, USA). A dilution of 1:1,000 is used for primary antibodies and 1:10,000 or secondary antibodies.
Validation	All antibody used are commercial and were validated

ChIP-seq

Data deposition

☒ Confirm that both raw and final processed data have been deposited in a public database such as GEO.

☐ Confirm that you have deposited or provided access to graph files (e.g. BED files) for the called peaks.

Data access links<br>*May remain private before publication.*	The ChIP-Seq data have been deposited in the NCBI BioProject database with accession code GSE213962 (https://www.ncbi.nlm.nih.gov/geo/query/acc.cgi?acc=GSE213962).
Files in database submission	ChIP-seq in the PH-1 _Input1.fq.gz<br>ChIP-seq in the PH-1_Input2.fq.gz<br>ChIP-seq of H3K4me3_ip1.fq.gz<br>ChIP-seq of H3K4me3_ip2.fq.gz<br>ChIP-seq of H3K27me3_ip1.fq.gz<br>ChIP-seq of H3K27me3_ip2.fq.gz<br>ChIP-seq of H3K4me3-H3K27me3_ip1.fq.gz<br>ChIP-seq of H3K4me3-H3K27me3_ip2.fq.gz<br>ChIP-seq of H3K27me3-H3K4me3_ip1.fq.gz<br>ChIP-seq of H3K27me3-H3K4me3_ip2.fq.gz<br>ChIP-seq in the PH-1 infection_input1.fq.gz<br>ChIP-seq in the PH-1 infection_input2.fq.gz<br>ChIP-seq of H3K4me3 in the PH-1 infection_ip1.fq.gz<br>ChIP-seq of H3K4me3 in the PH-1 infection_ip2.fq.gz<br>ChIP-seq of H3K27me3 in the PH-1 infection_ip1.fq.gz<br>ChIP-seq of H3K27me3 in the PH-1 infection_ip2.fq.gz<br>ChIP-seq in the PH-1 infection sequential ChIP_input1.fq.gz<br>ChIP-seq in the PH-1 infection sequential ChIP_input2.fq.gz<br>ChIP-seq of H3K4me3-H3K27me3 in the PH-1 infection_ip1.fq.gz<br>ChIP-seq of H3K4me3-H3K27me3 in the PH-1 infection_ip2.fq.gz<br>ChIP-seq of H3K27me3-H3K4me3 in the PH-1 infection_ip1.fq.gz<br>ChIP-seq of H3K27me3-H3K4me3 in the PH-1 infection_ip2.fq.gz
Genome browser session<br>(e.g. UCSC)	F. graminearum strain PH-1 GCA_900044135.1

Methodology

Replicates	Each ChIP-seq sample has two replicates.
Sequencing depth	Each experiment sequence 20M reads,single-end,length of reads is 50bp.
Antibodies	anti-H3K27me3 antibody (Thermofisher, 39155, MA, USA; 1:500 dilution); anti-H3K4me3 antibody (Abcam, ab8580, Cambridge, UK; 1:500 dilution) were used in this study.
Peak calling parameters	MACS2 (version 2.1.4)was used to call peaks by following parameters: callpeak -nomodel -extsize 147 -gsize 36,458,000.
Data quality	Peaks with p-value < 0.05 were retained.
Software	Bowtie2 (version 2.4.5) ;MACS2(version 2.1.4);Deeptools(version 2.4.1);Integrative Genomics Viewer(IGV,version 2.8.9).
